# Temporal Controls of the Asymmetric Cell Division Cycle in *Caulobacter crescentus*

**DOI:** 10.1371/journal.pcbi.1000463

**Published:** 2009-08-14

**Authors:** Shenghua Li, Paul Brazhnik, Bruno Sobral, John J. Tyson

**Affiliations:** 1 Department of Biological Sciences, Virginia Polytechnic Institute and State University, Blacksburg, Virginia, United States of America; 2 Virginia Bioinformatics Institute, Virginia Polytechnic Institute and State University, Blacksburg, Virginia, United States of America; University of Virginia, United States of America

## Abstract

The asymmetric cell division cycle of *Caulobacter crescentus* is orchestrated by an elaborate gene-protein regulatory network, centered on three major control proteins, DnaA, GcrA and CtrA. The regulatory network is cast into a quantitative computational model to investigate in a systematic fashion how these three proteins control the relevant genetic, biochemical and physiological properties of proliferating bacteria. Different controls for both swarmer and stalked cell cycles are represented in the mathematical scheme. The model is validated against observed phenotypes of wild-type cells and relevant mutants, and it predicts the phenotypes of novel mutants and of known mutants under novel experimental conditions. Because the cell cycle control proteins of *Caulobacter* are conserved across many species of alpha-proteobacteria, the model we are proposing here may be applicable to other genera of importance to agriculture and medicine (e.g., *Rhizobium*, *Brucella*).

## Author Summary

Because of its small genome size and the ease by which it can be manipulated genetically and biochemically, *Caulobacter crescentus* provides unique opportunities to study the molecular circuitry controlling the asymmetric cell division cycle of bacteria. A large amount of experimental data accumulated on this model organism in recent years needs to be quantitatively reconciled and analyzed in order to generate a full description of the process. Here, from these experimental clues, we suggest a mechanism for the principal molecular interactions that control DNA synthesis and asymmetric cell division in *Caulobacter* and construct a quantitative (mathematical) model of the mechanism in order to analyze the temporal dynamics of the control system. The model is centered around three “master regulator” proteins, whose timing of expression is tightly controlled by the progression of DNA replication. The model has been validated against observed phenotypes of wild-type cells and relevant mutants, and predicts phenotypes of novel mutants and of known mutants under novel experimental conditions. It provides a rigorous account of current intuitive ideas of bacterial cell cycle control and advances our understanding of bacterial cell division.

## Introduction

Understanding how cell division is controlled by underlying networks of interacting genes and proteins is of fundamental importance to the life sciences, the biotech industry and human/veterinary medicine. Theoretical biologists have vigorously pursued the quantitative analysis of cell cycle controls in eukaryotes, e.g., in yeast [1]–[4], in frog eggs [5],[6], in fruit flies [7], and in mammalian cells [8]–[13], but similar studies of cell cycle regulation in prokaryotes have lagged behind. Since the early era (1980–1991) of mathematical modeling of the initiation of DNA replication in *Escherichia coli* [14]–[20], there have been few theoretical studies of cell cycle control in bacteria [21]–[23] until the recent appearance of two papers on the molecular regulation of DNA replication and cell division in *Caulobacter crescentus* [24],[25]. *Caulobacter* has become a model organism for genetic analysis of prokaryotic cell cycle regulation [26], and the resulting wealth of molecular details provides fertile ground for computational modeling that is realistic, comprehensive and predictive.

**Figure 1:**
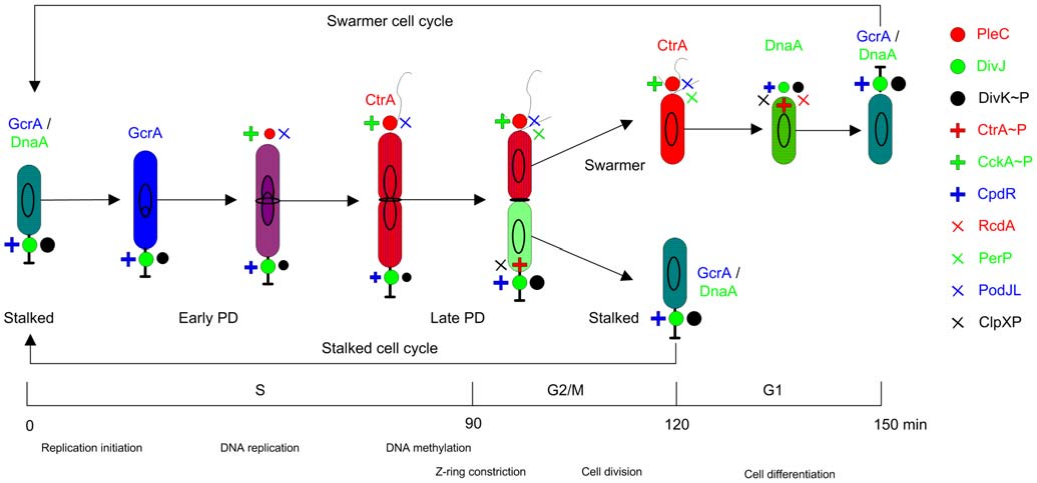
Physiology of the cell division cycle in *Caulobacter crescentus.* Three cell cycle phases can be distinguished (from the left to the right): a DNA synthesis (S) phase that takes approximately 90 min, a cell division (G2/M) phase, lasting approximately 30 min, that culminates in the separation of mother (stalked) and daughter (swarmer) cells, and a growth and differentiation (G1-like) phase of the swarmer cell that lasts approximately 30 min. The color scheme denotes protein variations through the cell division cycle: GcrA (blue), CtrA (red), DnaA (green). The θ-like structure denotes replicating DNA. The ring in the middle of the cell indicates Z-ring assembly and constriction, leading to cell separation (cytokinesis). Symbols of different shapes at the two cell poles denote localization of proteins. Their identities are listed at the right of this figure. PD, predivisional. At the bottom, the time scale and important cell cycle-related physiological events are indicated.

The gram-negative, aquatic alpha-proteobacterium, *Caulobacter crescentus* undergoes asymmetric division producing two progeny cells with identical genome but different developmental programs: the sessile “stalked” cell immediately enters another replication cycle, whereas the flagellated “swarmer” cell swims away before differentiating into the staked morphology and re-entering the division cycle (Figure 1) [27]–[29]. In Figure 1 we refer to the phases of the *Caulobacter* cell cycle as G1, S and G2/M, as is common terminology among *Caulobacter* researchers. The more common convention among bacteriologists is “C and D stages” (C = stage of DNA replication, D = stage from termination of DNA synthesis to cell division). The C/D distinction is convenient for rapidly growing bacteria with overlapping rounds of DNA replication. Slowly growing bacteria, like *Caulobacter*, can be said to have a B period (stage of unreplicated DNA), but we prefer to use the “eukaryotic” terminology of G1, S and G2/M phases because of the striking similarities between the cell cycles of *Caulobacter* and budding yeast [23],[30]

Cell division cycles of swarmer and stalked cells share the same core regulatory system that controls the cell's commitment to a new round of DNA synthesis and to the asymmetric division process [31]. Two proteins (DnaA and GcrA) in particular control the onset of DNA replication, and a third master regulator (CtrA) controls cell division and cell fate events through a complex network of protein phosphorylation and degradation reactions (involving PleC, DivJ, PodJ, PerP, CckA, DivK and other components).

Stalked cells alternate periodically between the DNA synthesis phase (S) and the cell division phase (G2/M). Swarmer cells exhibit, in addition, a G1-like phase during which the bacterium grows and moves around, before differentiating into a stalked cell and entering into S phase. The molecular mechanism guiding a swarmer cell through G1 phase has not yet been completely worked out. But experimental evidences suggest that PodJ, PleC, and DivJ proteins regulate the phosphorylation state of DivK, which in turn controls the abundance of CtrA in the swarmer cell (high CtrA level blocks entry into S phase) [32]–[34]. Incorporating this idea into our previous model of stalked cell cycle control, we have created a mathematical model (in terms of ordinary differential equations) that allows us to investigate the temporal dynamics of these regulatory proteins and their associated physiological events in both stalked and swarmer cells. The model provides a rigorous account of the consequences of our hypotheses, which can be compared to experimental observations to test the model.

In this new version of our model we also incorporate explicitly the phosphorylation of CtrA and its regulation by CckA [35],[36], which allows us to capture the behavior of double mutants reported in [37]. Proteolysis of CtrA has also been refined to include recently described effects of RcdA and CpdR [35],[38]. Finally, regulation of cytokinesis via the Z-ring has been redesigned to include the FtsZ and FtsQ/A proteins, a phenomenological variable describing formation of the Z-ring, and a checkpoint signal from ongoing DNA replication via the ParA pathway [39]. As for previous model, we do not account explicitly for spatial localization of proteins, leaving this aspect of the control system for later versions.

### A Consensus Picture of Cell Cycle Controls in *C. crescentus*

The molecular network relevant to our model of the stalked cell division cycle of *Caulobacter* has been described in great detail previously [25], so here we describe only those new components of the model involved in the swarmed-to-stalked cell transition, and some details related to improvements of our core model.

#### CtrA production, phosphorylation and proteolysis

The relative abundance of CtrA in a daughter cell right after division determines the morphology of the cell: CtrA is elevated in the incipient swarmer cell, but it is degraded to very low level in the stalked cell compartment. At some later time, CtrA must be degraded in the swarmer cell during its transition to the stalked morphology. The level of CtrA in the cell is determined by the balance between protein synthesis and degradation, which processes are regulated in turn by the actions of other proteins. Production of CtrA is initiated by GcrA [40], promoted by CtrA itself [41], and is a subject to the methylation state of the *ctrA* gene [42].

Once synthesized, CtrA protein must be phosphorylated into an active form, CtrA∼P, to perform its functions [29]. During the division cycle of wild type cells, CtrA∼P follows closely the time course of CtrA (with less than 10 min delay) [27],[29],[31],[40], in some mutant cells this synchrony is lost [36],[37].

The CckA histidine kinase contributes to CtrA phosphorylation. The *cckA* gene is expressed only briefly during the swarmer-to-stalked cell transition [36]. Otherwise, the total CckA level is stable throughout the cell cycle [43]. CckA molecules become localized and phosphorylated during the cell cycle. When phosphorylated, CckA∼P activates CtrA phosphorylation [29]. During the cell cycle, CckA∼P concentration is tightly correlated with CtrA∼P concentration (they show similar pattern of variation) [36],[44]. Because CckA contributes to the phosphorylation of several cell-cycle control proteins, there may be competition for phosphate groups among its targets. (We do not attempt to model such effects at this time.) In addition, DivL is reportedly involved, to some extent, in the process of CtrA phosphorylation [29],[45].

Degradation of CtrA and its phosphorylated form, CtrA∼P, is also regulated. The phosphorylated protein, DivK∼P, accelerates proteolysis of both forms of CtrA [46]. This effect was used in our previous model to define the time when CtrA starts being degraded. The mechanism of CtrA proteolysis is complicated. The protease ClpXP is directly involved in degrading CtrA and CtrA∼P, with the help of another protein, RcdA, whose production is activated by CtrA∼P [35],[47]. Although ClpXP is stable throughout the cell cycle [48], even though its promoter activity varies [49], the localization of ClpXP is regulated by the CpdR protein. When CpdR is dephosphorylated and localized, it recruits ClpXP to the cell poles where CtrA/CtrA∼P is degraded. Thus, the activation of CpdR by dephosphorylation can be viewed as another determining factor for CtrA and CtrA∼P degradation [38].

Finally, it needs to be mentioned that the pathways of CtrA phosphorylation and proteolysis are interwoven. High DivK∼P represses CckA phosphorylation [50], thus down-regulating indirectly CtrA phosphorylation and CtrA activity. CckA∼P activates CpdR phosphorylation and affects CpdR localization, which represses indirectly the degradation CtrA and CtrA∼P [35],[50].

#### PleC and DivJ roles in DivK phosphorylation

The histidine kinases PleC and DivJ are two proteins that facilitate dephosphorylation and phosphorylation of DivK respectively, during the cell cycle. According to microarray data, *pleC* expression is activated by GcrA [40], but total PleC protein level is relatively stable during the cell cycle [34]. The protein localizes to the flagellar cell pole once expressed. Localization of the protein is required for its function, and the full length PodJ_L_ affects PleC localization [51].

Expression of the polar organelle development gene *podJ* is upregulated by GcrA and DnaA and downregulated by CtrA∼P [31],[40],[41],[52],[53]. The PodJ protein has two distinct isoforms: the full-length translation product PodJ_L_ and a truncated form PodJ_S_. PodJ_L_ localizes to the incipient swarmer pole, where it helps to recruit factors required for polar morphogenesis, including PleC. The periplasmic domain of PodJ_L_ is degraded by a periplasmic protease PerP, giving rise to a truncated form of the protein PodJ_S_. Lower abundance of PodJ_L_ leads to the release of PleC from flagellar cell pole [34]. During the swarmer-to-stalked cell transition, PodJ_S_ is cleared and PodJ_L_ is synthesized and localized again [51].

The protease PerP is required for efficient truncation of PodJ_L_. Microarray analysis shows that *perP* expression is activated by CtrA∼P [41],[54]. In addition, polar PleC activity also affects, to some extent, *perP* expression [55].

Therefore, PodJ_L_, PerP and PleC work together in regulating the truncation of PodJ_L_ and consequent release of PleC [55]. This network dynamically regulates the temporal distributions of PodJ_L_ and PodJ_S_, and the spatial localization of PleC. PleC, in turn, activates the dephosphorylation of DivK in the swarmer and predivisional cells through the cell division cycle [51].

DivJ is present at low concentration in a swarmer cell but increases dramatically during the swarmer-to-stalked transition [33]. Then, it localizes to the stalked cell pole and stays steady during the rest of the cell cycle. DivJ's localization helps the phosphorylation of DivK. PleC directly or indirectly regulates localization of DivJ [33],[56]. The SpmX protein was recently reported as a factor involved in the process of DivJ localization after the release of PleC from the flagellar cell pole [57]. Otherwise, little is known about the regulation of DivJ in cells. High localized DivJ functions as a kinase in the stalked cell, where it activates the phosphorylation of DivK. In addition, recent data show that DivL is involved in the phosphorylation of DivK, directly or indirectly [58].

Because of the opposite localization of PleC and DivJ in a predivisional cell, a concentration gradient of DivK phosphorylation state is formed in predivisional cells, with DivK predominant at the swarmer pole and DivK∼P predominant at the stalked pole [33],[57]. After division, PleC and DivJ are separated to different compartments (nascent cells). Therefore, in the nascent swarmer cell, DivK gets quickly dephosphorylated (driven by PleC), which preserves a high level of CtrA in this compartment. In the nascent stalked cells, all DivK becomes phosphorylated due to the action of DivJ, which accelerates the degradation of CtrA. During the swarmer-to-stalked cell transition (G1/S transition), release of PleC and consequent localization of DivJ at the same pole activates DivK phosphorylation [57] leading to rapid degradation of CtrA.

#### Z-ring constriction, coupled with DNA replication

Cytokinesis in *Caulobacter* is regulated by a number of factors, including DNA replication and segregation, flagellum assembly, and the master regulators CtrA and DnaA [53],[59].

CtrA∼P regulates transcription of a number of proteins involved in the formation and closure of the septal Z-ring, including *ftsZ*, *ftsQ*, *ftsA* [60]–[62]. In a predivisional cell and in a swarmer cell, CtrA∼P represses transcription of *ftsZ*, the gene whose product (bacterial counterpart of tubulin) is a building block of the Z-ring [62]. DnaA, on the other hand, is an activator for *ftsZ* expression [53]. FtsZ was measured at maximal level in an early predivisional cell that has a visible Z-ring, then it decreased as CtrA∼P level rises [61]. Following the assembly of the Z-ring, *ftsQ* and *ftsA* are activated by CtrA∼P in an orderly manner [61]. Importantly, FtsQ, a regulator of Z-ring constriction, is only produced in predivisional cell phase when DNA is being replicated [63]. It is not produced in a swarmer cell even though CtrA∼P is high in this stage of the cell cycle.

A short DNA sequence, *parS*, locates close to the origin of replication (*C_ori_*). The chromosome partitioning protein, ParB, binds to *parS* and colocalizes with the origin of replication at the flagellated pole in swarmer cells [64]–[66]. Soon after the initiation of DNA replication, one copy of the origin moves to the opposite pole of cell and, as a result, ParB starts to exhibit a bipolar localization pattern. Another chromosome partitioning protein, ParA, also shows a bipolar localization pattern in the predivisional cell and may form a complex with ParB at the origin. Depletion of ParB or overexpression of ParA results in filamentous cells that lack Z-rings or form them in improper locations [67]. Total amounts of ParA and ParB stay constant during the cell cycle [68]. Correct localization of these proteins is a necessary condition for correctly positioning the Z-ring in the middle of a cell [67]. Moreover, nucleotide exchange between ParA-ATP and ParA-ADP is regulated by ParB [69]. An increased level of ParA-ADP (which is proposed to be regulated by high ParB in the cytoplasm) inhibits Z-ring assembly and cell division [69]. It has been suggested that ParA-ADP is the active form of ParA that inhibits cell division by preventing the formation and constriction of the Z-ring, directly or indirectly [67]. In summary, DNA replication and chromosome partitioning accompanied by ParB and ParA rearrangement results in a lower level of ParB and ParA-ADP in the cytoplasm, which releases the repression of Z-ring formation and allows Z-ring constriction in the center of the cell.

In addition, CtrA∼P activates class II genes for flagellum assembly, including σ^54^ [35],[59]. The flagellar regulatory protein FlbD is activated by σ^54^, which lead to the activation of a gene required for optimal FtsZ ring assembly. Recently, in *Caulobacter*, a spatial regulator coordinating chromosome segregation, MipZ, was recognized as an element with functions similar to those of the MinCDE system in *E.coli* (which serves to repress Z-ring formation and constriction) [39],[70]. MipZ, activated by DnaA [39],[71], is also involved in communication between ParB and Z-ring assembly and constriction [47]. Thus, Z-ring assembly and constriction are elaborately regulated, directly or indirectly, by DnaA and CtrA∼P proteins, even though the details of how it happens in the cell are as yet unclear.

## Results

**Figure 2:**
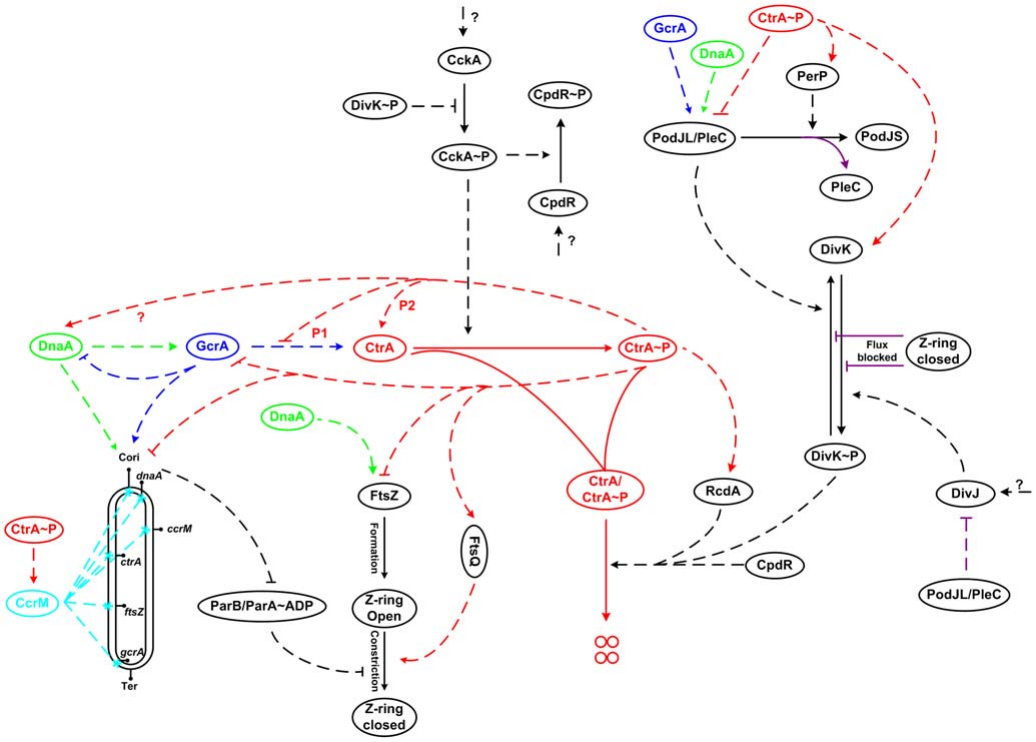
Wiring diagram of the model. All proteins (ovals) are assumed to be produced and degraded at specific rates. Only degradation of CtrA and CtrA∼P is depicted explicitly (4 small circles indicate products of degradation), in order to show how these steps are regulated. Solid lines correspond to the mass flow while dashed lines denote regulatory effects. P1 and P2 denote the two promoters controlling CtrA production. Purple lines signify the role of localization/delocalization effects in corresponding regulations. The double-stranded closed curve at the bottom left represents DNA. *C_ori_* is the origin of DNA replication and *Ter* stands for the termination site. DNA methylation sites on genes are marked by cyan stars. Z-ring closure at the far right blocks the flux of DivK and DivK∼P between swarmer and stalked cell compartments.

Figure 2 presents an informal wiring diagram of the molecular interactions captured in our model. The corresponding mathematical model is given in Supplemental Table S1 and outlined in Materials and Methods. A detailed description of our model can be found on our Web site (http://mpf.biol.vt.edu/research/caulobacter/SWST/pp/), including a machine-readable version of the model (see also, Text S2). To simulate the molecular regulation of the cell division cycle, we solve the equations in Table S1 subject to the parameter values and initial conditions in Tables S2 and S3.

### The Model Correctly Represents the Sequence of Physiological Events during Asymmetric Cell Division in *C. crescentus*

**Figure 3:**
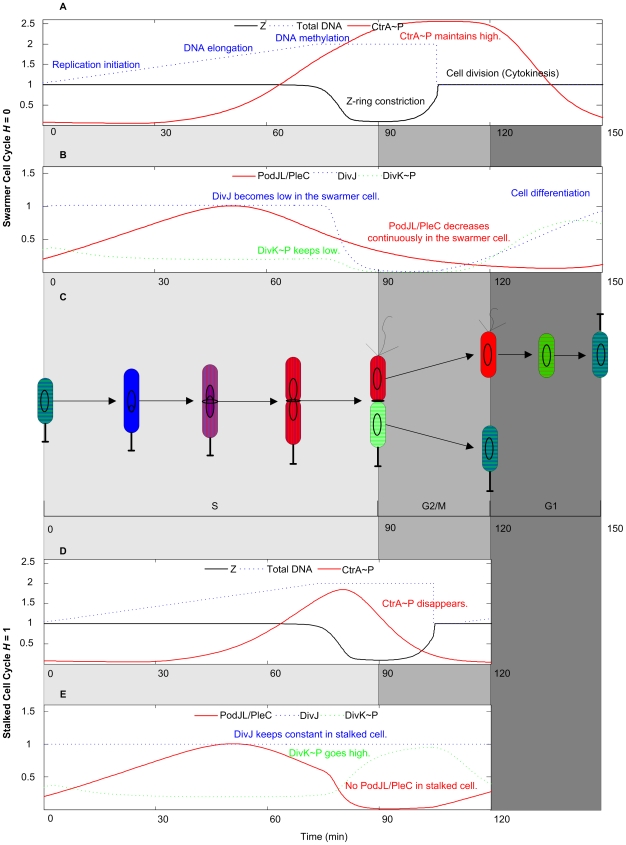
Correlations between protein levels and the physiological features of a dividing *Caulobacter* cell. Time courses of protein expressions, DNA replication and Z-ring states produced by our model for the swarmer cell cycle (A, B) and for the stalked cell cycle (D, E). Physiological changes during cell cycle progression are shown in panel C.

The temporal sequence of physiological events during both the stalked cell and the swarmer cell cycle is correctly reproduced by the model. (In our simulations, we track either the swarmer, SW, or the stalked, ST, cell cycle by setting a parameter called *H* to 1 for ST or to 0 for SW.) After the initiation of DNA synthesis (Figure 3, at t = 0 in both stalked and swarmer cell cycles), the DNA molecule is progressively replicated and then methylated over a period of ∼90 minutes. The molecular components necessary for cell division (CtrA∼P, PodJ_L_/PleC, DivJ, DivK∼P) are expressed in a timely fashion during S phase (Figure 3). At the end of S phase, the Z-ring starts to constrict (as described by the variable [Z] in Eq. 28 of Table S1), and it takes about 15 min to finish this process (Figure 3A&D). After that, the predivisional cell enters into G2/M phase during which cell division (cytokinesis) begins, and the swarmer and stalked cell compartments embark on different developmental programs. During 30 min of G2/M phase, PodJ_L_/PleC and DivJ are completely separated into the swarmer and stalked cell compartments respectively (Figure 3B&E) due to the constriction of the Z-ring (i.e., as [Z] falls from 1 to 0). During G2/M phase, the regulatory proteins PodJ_L_/PleC, DivJ, and DivK∼P take on different values in our simulated swarmer and stalked cell compartments (Figure 3A, B, D &E). These differences lead to different developmental tracks of the two progeny cells.

After 30 min of G2/M phase, the progeny stalked cell is ready (because of its low level of CtrA∼P, see Figure 3E) to enter another cell cycle. The progeny swarmer cell, however, enters into a G1-like phase which lasts about 30 min, as experimentally observed. During G1 phase, our simulation shows how the proteins (PodJ_L/_PleC, DivJ, DivK/DivK∼P and CtrA/CtrA∼P in Figure 3) change with time to convert the swarmer cell back to a stalked cell, ready to enter another cell cycle.

In a summary, the timing of such major events of the cell cycle as DNA replication, Z-ring assembly and constriction, cytokinesis, and cell differentiation agrees well with experimental observations [26],[27],[29],[31],[72].

### The Model Accurately Describes Protein Expression Patterns during the Division Cycles of both Stalked and Swarmer Wild-Type Cells

**Figure 4:**
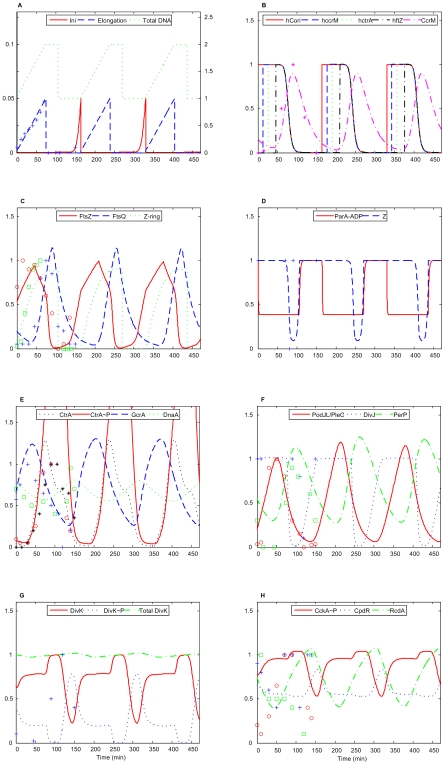
Simulated variations of proteins and other model state variables during the swarmer wild-type cell cycle. Simulation begins with initiation of DNA replication. Three cell cycles are shown. Experimental data presented on some panels by open circles and squares, crosses and asterisks are re-plotted from the following sources: (A) Total DNA from Figure 4 in [84]. (B) CcrM from Figure 2 in [85]. (C) FtsZ from Fig. 2C in [86]; Zring from Fig. 3C in [87]; FtsQ from figure 2 in [86]. (D) Z from Figure 2 in [88]. (E) CtrA from Fig. 3C in [40]; CtrA∼P from Fig. 3C in [36]; GcrA from Figure 3 in [40]; DnaA from Figure 5 in [89]. (F) PodJL/PleC from Fig. 1A in [55] and Fig. 1B in [34]; DivJ from Fig. 2B in [33]; PerP from Fig. 4 in [55]. (G) DivK∼P from Figure 2 in [85]. (H) CckA∼P from Fig. 3B in [36]; CpdR from Fig. 5A in [35]; RcdA from Fig. 2C in [38].

Figure 4 provides more details about variations of proteins included in the model during the division cycle of a swarmer cell. The swarmer cell cycle is identical to the stalked cell cycle from the initiation of DNA synthesis (at t = 0, Figure 4A&B) until the Z-ring constriction (at t = 90 min, see Figure 4C). At this period of time, DivJ is located at the stalked cell pole and PodJ_L_/PleC at the swarmer cell pole (Figure 4F). Because DivK has access to both PodJ_L_/PleC and DivJ in the pre-divisional cell, the level of DivK∼P is very low at the onset of Z-ring constriction (Figure 4G).

During G2/M phase (90∼120 min in Figure 4), the Z-ring assembles and constricts (Figure 4C&D) in about 15 min, and total DNA divides (Figure 4A) evenly between the two progeny cells. PodJ_L_/PleC is separated into the swarmer cell compartment (Figure 4F) while DivJ remains behind in the stalked cell compartment. Therefore, DivK∼P drops sharply in the incipient swarmer cell (70–100 min in Figure 4G). With DivK∼P level low and CckA∼P level high (Figure 4H) in the incipient swarmer cell, CtrA∼P is maintained at a high level of activity (90∼120 min in Figure 4E).

During the swarmer-to-stalked cell transition (120∼150 min in Figure 4), PodJ_L_/PleC plummets and DivJ becomes active again (Figure 4F); hence, DivK becomes phosphorylated and CtrA∼P is rapidly degraded (Figure 4E). GcrA and DnaA levels climb (Figure 4E), and the new stalked cell is ready for a new round of DNA replication.

In some panels in Figure 4 (see also Figure S2), we compare our simulation with experimental data, collected from many different publications. The protein variations are given in relative units. In most cases, experimental uncertainties were not reported, but it is reasonable to assume that the error bounds should be quite generous. The data sets provide us with qualitative benchmarks for our simulations, but a quantitative assessment of goodness-of-fit would be inappropriate. Based on visual comparison, we conclude that our simulated protein variations agree reasonably well with the experimentally observed patterns.

### The Model Agrees with the Phenotypes of Mutant Strains

Mutant cells provide valuable information about how individual components of the cell cycle control system affect phenotypes of cells. We simulate mutants using exactly the same differential equations, parameter values, and initial conditions as for wild type (*wt*) cells (Tables, S1, S2, and S3), except for those modifications to parameters dictated by the mutation (Table S4). For instance, a mutant with a particular gene deleted is modeled by zeroing the production rate of the corresponding protein. This change propagates through the network, modifying the time courses of other proteins, and from these profiles we infer the phenotype of the mutant.

Here we present our simulations for *ctrAD51E*, *ctrAΔ3*Ω, *ctrAD51EΔ3*Ω, *ctrA* constitutive expression, *ctrA^op^*, *ΔdivJ*, and *ΔpleC*/*ΔpodJ* mutants. The first five mutants were not correctly simulated in our earlier model because we made no distinction between phosphorylated (active) and unphosphorylated (inactive) CtrA. The present model now accounts for the observed phenotypes of these *ctrA* mutants. The other two mutants, *ΔdivJ* and *ΔpleC*/*ΔpodJ*, were outside the scope of our earlier model. All other mutants that we have simulated are presented in the website http://mpf.biol.vt.edu/research/caulobacter/SWST/pp/.

#### *ctrAD51E*

In this mutant the CtrA phosphorylation site, aspartate 51, is replaced with a glutamate residue. This creates a form of the CtrA which cannot be phosphorylated but is, nevertheless, able to activate downstream genes [73]. When the *ctrAD51E* gene was expressed from a high-copy number plasmid in cells deleted for the *wt* chromosomal *ctrA* gene, the mutated cells were observed to have normal morphology and DNA content (Figure 5 in [37]).

**Figure 5:**
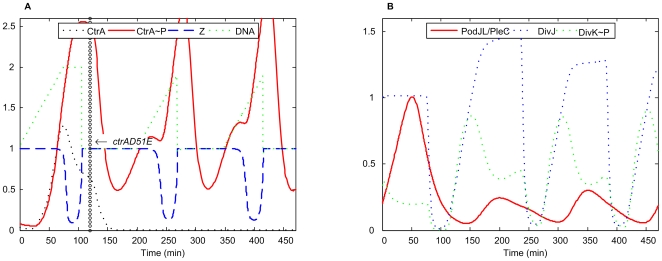
Simulation of *ctrAD51E* mutant. *k*_s,ctrA-P1_ = *k*_s,ctrA-P2_ = 0, *k*_trans,CtrA∼P_ = 0, *k* ′ = 0.064 (40% of WT) was added to [CtrA∼P] equation. The vertical column of open circles here and on subsequent figures indicates the time at which the mutation is introduced. For earlier times the simulation is run with wild-type values of all parameters.

Since in this mutant the constitutively expressed CtrA is active all the time, we simulated the mutant by turning off CtrA production from the original gene and by producing the active form of CtrA (phophorylated form in our model) constitutively. Our simulation shows (Figure 5) that an elevated level of CtrA∼P does not block progression of cells through the cell cycle. The CtrA degradation machinery is able to lower the level of CtrA∼P enough that the necessary conditions for DNA replication are satisfied, while the components for the Z-ring assembly and constriction are also available when needed. Elevated CtrA∼P reduces to some extent GcrA, PodJ_L_/PleC, FtsZ and Zring, and accelerates DNA methylation, but these changes do not have lethal effects on cell cycle progression.

It can be observed from our simulation (Figure 5) that Z-ring closing time is a bit longer in the mutant than in *wt* cells. We consider this an artifact of our model, due to the oversimplified representation of the role of FtsQ. In our model, FtsQ abundance controls both the onset and duration of Z-ring constriction, while in reality this dual role may not be the case. (This artifact may also appear in other mutants.)

#### *ctrAΔ3*Ω

When the final three amino acids of CtrA are deleted (or substituted with a peptide tag), the rate of CtrA degradation is decreased to ∼10% of *wt* rate [37]. Nonetheless, these mutant cells exhibit normal cellular morphology and DNA content (Figure 5 in [37]).

**Figure 6:**
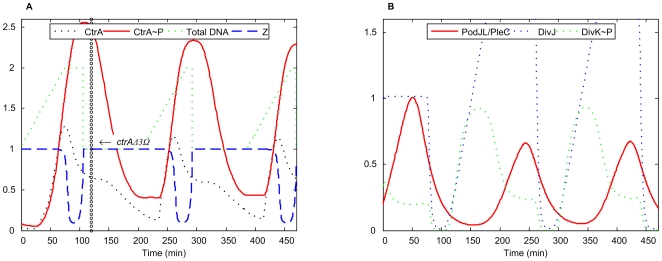
Simulation of *ctrAΔ3*Ω mutant. *k*_d,ctrA2_ = 0.0375 (15% of WT).

In our simulation of this mutant (Figure 6), CtrA and CtrA∼P fluctuate in a manner similar to *wt* cells. Notice that the steady state levels of CtrA and CtrA∼P during DNA synthesis phase are about 10-fold higher in the mutant than in *wt* cells because the degradation rate of CtrA (and CtrA∼P) protein 10-fold smaller in the mutants. Nevertheless, CtrA proteolysis is still sufficient to permit DNA synthesis. Later in the cell cycle, during the ∼50 min when the *ctrA* gene is hemimethylated and CtrA proteolysis is minimal, both *wt* cells and mutant cells accumulate nearly the same amount of CtrA and CtrA∼P. Hence, the mutant cell undergoes normal division cycle.

#### *ctrAD51EΔ3*Ω

**Figure 7:**
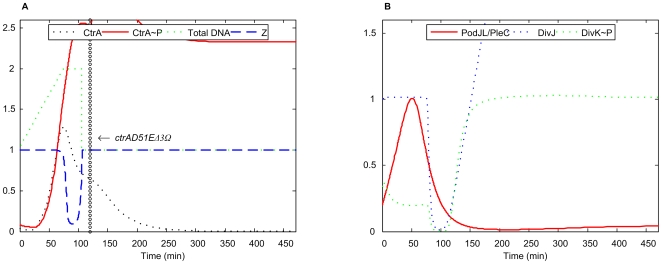
Simulation of double mutant *ctrAD51EΔ3*Ω. *k*_s,ctrA-P1_ = *k*_s,ctrA-P2_ = 0, *k*_trans,CtrA∼P_ = 0, *k* ′ = 0.064 (40% of WT) was added to [CtrA∼P] equation, *k*_d,ctrA2_ = 0.0375 (15% of WT).

This double mutant, which combines the properties of *ctrAD51E* and *ctrAΔ3*Ω, is observed to be filamentous and arrested in G1 phase [37]. In our simulation (Figure 7), CtrA∼P rises to such a high level that DNA replication cannot be initiated, consistent with G1 arrest and a filamentous morphology. In addition, based on the variation patterns of DivJ and DivK∼P, our model predicts that, after the introduction of mutation, the swarmer cell will differentiate into a stalked cell and then become arrested without initiation of DNA replication.

#### Constitutive *ctrA* expression

**Figure 8:**
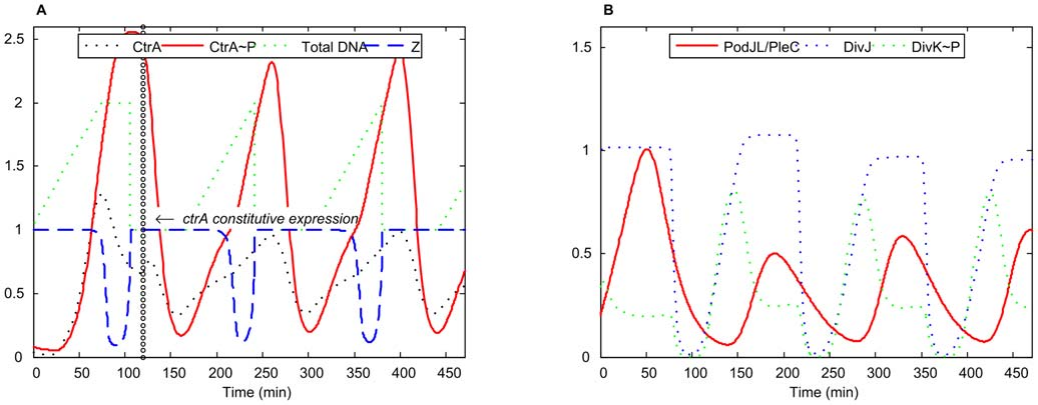
Simulation of *ctrA* constitutive expression. *ctrA* is constitutively expressed at 30% of its wild-type promoter activity: *k*_s,ctrA-P1_ = *k*_s,ctrA-P2_ = 0, *k* ′ = 0.048.

When *ctrA* is constitutively expressed (the only copy of *ctrA* gene is on pJS14), the cell cycle is normal [37]. In our simulation (Figure 8), constitutive *ctrA* expression at mild level (20%∼80% of wild-type *ctrA* promoter activity) does not affect the normal cell cycle. Insignificant deviations, similar to those for the *ctrAD51E* mutant, were observed for some proteins, Z-ring closing time, and DNA methylation. If *ctrA* is expressed constitutively at <20% of the wild-type level, then CtrA∼P never increases high enough to prompt expression of other essential genes, and the cell cannot proceed through the division cycle normally. The simulation results for this case are similar to those of *ΔctrA* mutant (data not shown here; see figure at the website http://mpf.biol.vt.edu/research/caulobacter/SWST/pp/).

If *ctrA* is expressed constitutively at too high level (above 80% of wild-type *ctrA* promoter activity), then CtrA∼P reaches quite a high level even though the proteolysis pathway works normally. The high level of CtrA∼P restricts the initiation of DNA replication and it cannot start at a right time. The cell cycle thus cannot proceed normally in this case. In [37], *ctrA* is expressed from a strong promoter, but the extent of overproduction is unknown. Our model predicts that the level of CtrA protein in this viable mutant should be in the range 20–80% of the wild type level because, if *ctrA* is greatly overexpressed (as in the *ctrA^op^* mutant described below), then cell cycle progression is blocked.

#### *ctrA^op^*

When the genomic copy of the *ctrA* gene is missing a coding sequence for the last three amino acids (*ctrAΔ3Ω*), the resulted mutated CtrA protein is more stable [37]. When this gene is introduced in cells on a high copy-number plasmid (*ctrA^+^* (wild type)*+P_xylX_-ctrAΔ3*) and the cells are grown on 0.2% xylose to overexpress the stable (mutated) form of CtrA, then the mutant cells become filamentous and arrested either in G1 phase (unreplicated DNA) or in G2 phase (replicated DNA) [74].

**Figure 9:**
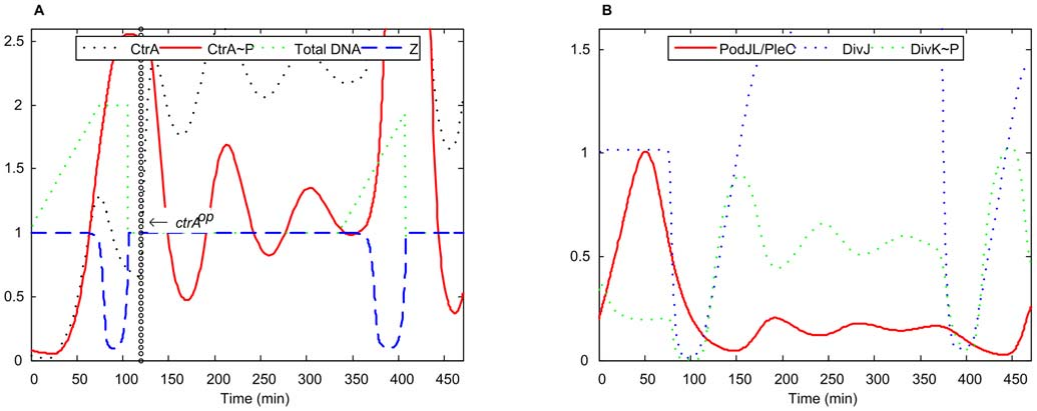
Simulation of *ctrA^op^* mutant. *k* ′ = 0.16.

In our simulation (Figure 9), elevated levels of DivJ and DivK∼P indicate that the swarmer cell may have differentiated into a stalked cell. However, the high level of CtrA∼P represses initiation of DNA replication in the mutant cell, making it arrest in G1 phase. The simulation for this case is similar to the case of *ctrA* constitutive expression (>80% of maximum *wt* rate), as just described. For the stalked cell, the division cycle can be arrested either in G1 phase or G2 phase, based on our simulation reported in [25].

After a longer duration (∼200 min in our simulation), however, a new round of DNA replication starts in our simulation. This is a side-effect of the overly simplified term in our model for the initiation of DNA replication.

#### *ΔdivJ*

*divJ^cs^* [75], *divJ_H338A_* [76], *ΔdivJ* [33], and *divJ:: Ω(Spc^R^)* [77] are all described as *divJ* deletion mutants in experiments. In these mutant cells, DivK∼P level is reduced and, as a result, cells contain several copies of chromosomes and became filamentous.

**Figure 10:**
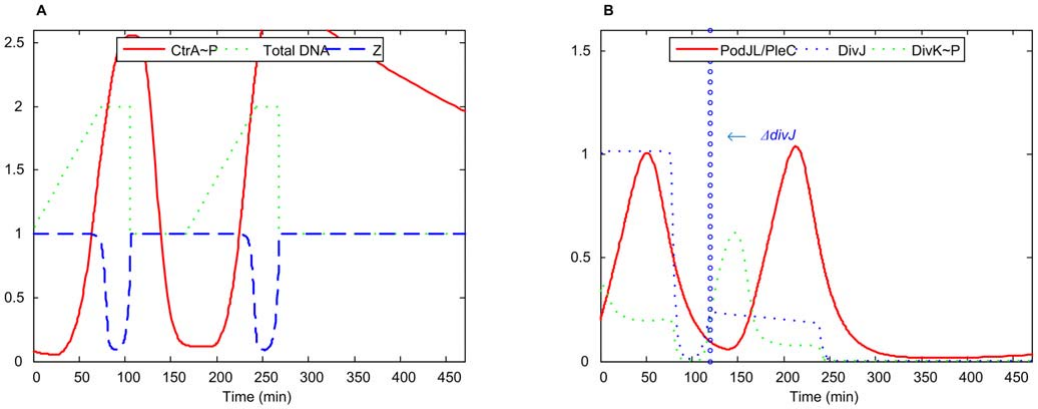
Simulation of *ΔdivJ* mutant. *k*_s,DivJ1_ = *k*_s,DivJ2_ = 0.

In our simulation of these mutants (Figure 10), DivK∼P level drops as expected, and CtrA∼P stays high, repressing a new round of DNA replication after cell division. The mutant cells arrest in G1 phase and become filamentous. Without DivJ and DivK∼P, the cell should remain in the swarmer cell morphology.

It has been reported that DivJ is also involved in repressing the initiation of DNA replication [33], a feature that is not implemented in our model. This may be the reason why we do not see, in our simulation, accumulation of multiple chromosomes, as was reported experimentally.

#### *ΔpleC* and *ΔpodJ*

Elevated levels of DivK∼P are observed in *ΔpleC* mutant cells (*pleC::Tn5*) [33],[75],[78],[79]. *ΔpodJ* mutant cells (*podJ::Tn5* or *podJ-xylX*) become filamentous, and their phenotype is similar to *ctrA401* cells [51],[80].

**Figure 11:**
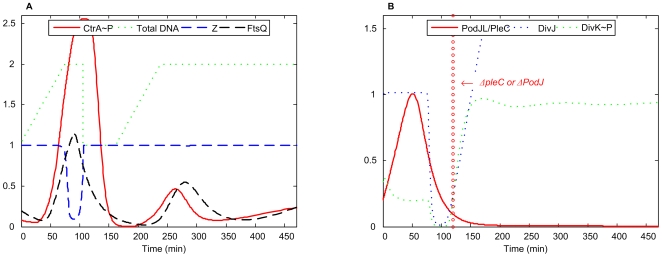
Simulation of *ΔpleC*/*ΔpodJ* mutants. *k*_s,PodJL_ = 0.

In our simulation (Figure 11), the lower level of PodJ_L_/PleC causes accumulation of DivK∼P, which in turn lowers CtrA∼P. Consequently, DivJ is elevated, which indicates (in our model) that the cell has differentiated into a stalked morphology. DNA replication could be initiated in this case but cell division does not proceed due to insufficient FtsQ (caused by low level of CtrA∼P). Thus, such a cell is arrested in G2 phase and become filamentous in the simulation.

### The Model Predicts Phenotypes of Novel Mutants

Predictions of the model provide for directions for designing new experiments, including direct experimental tests of the model. In each simulation of a known mutant genotype, described above, some of the results were compared against experimental observations, and other parts may be considered as predictions, because the experimental studies did not report relevant information (e.g. variations of some proteins). These quantitative predictions are useful for future experimental exploration of these mutants that have already been studied to some extent. In addition, we present in this section simulations of some novel mutants that have not been described in the literature, to our knowledge. Some other novel mutants are also described at our website http://mpf.biol.vt.edu/research/caulobacter/SWST/pp/.

#### *pleC^op^* and *podJ^op^*

**Figure 12:**
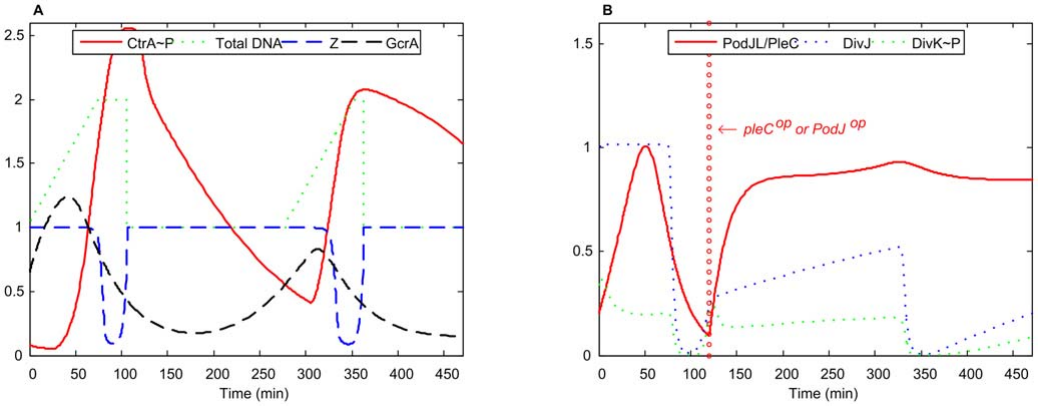
Simulation of *pleC^op^/podJ^op^* mutant. *k*_s,PodJL_ = 0, *k*′ = 0.043 (100% of WT).

The effects of overexpressing PleC or PodJ have not been reported in the literature. In our simulation of these mutants (Figure 12), the high level of PodJ_L_/PleC leads to an elevated level of CtrA∼P. If DNA replication has been initiated by this time, the cell may proceed successfully through another division cycle (as in Figure 12) despite a high level of CtrA∼P. However, elevated CtrA∼P decreases the levels of GcrA, DivK∼P and DivJ in our simulation, which may cause problems for these mutant cells in subsequent generations. Cells may arrest in G1 with low GcrA, or they may proceed through another round of DNA replication and arrest in G2 with high CtrA∼P. The expected phenotype depends sensitively on parameter values in the model and cannot be predicted precisely.

#### CckA unphosphorylated

**Figure 13:**
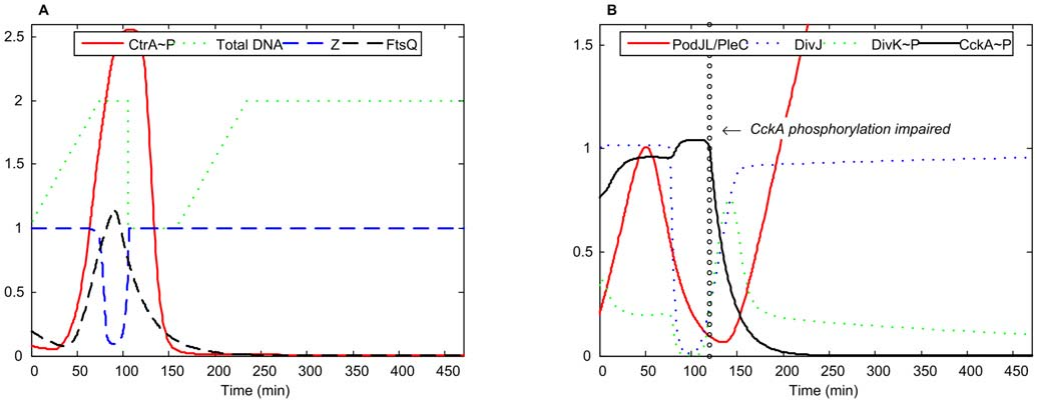
Simulation of unphosphorylated CckA mutant. *k*_trans,CckA_ = 0.

If CckA cannot be phosphorylated, then, according to our simulation (Figure 13), CtrA∼P level will fall. As a result, DivJ rises, indicating that the cell has transformed into a stalked morphology, and a new round of DNA replication is initiated, but the Z-ring cannot constrict due to insufficient FtsQ. Thus our simulation for this mutant indicates that the cell is arrested in the G2 phase with replicated chromosomes and filamentous morphology.

### Website of the Model and Online Simulator

To organize all simulations (for wild-type cells and for mutants) and present them in a systematic way, we have developed a website (http://mpf.biol.vt.edu/research/caulobacter/SWST/pp/) that includes an introduction, a description of the mathematical model, wild-type simulation results, simulations of all relevant mutants, model files, modeling tools and an online simulator (http://mpf.biol.vt.edu/research/caulobacter/SWST/pp/onlinesimulator.php) to run the mathematical model. The web site is designed to help molecular biologists design new experiments and mathematical modeler to explore the model in greater detail. The online simulator has a friendly interface for running the model without getting unnecessarily involved in the underlying differential equations and parameter settings.

## Discussion

Molecular cell biologists have collected a large amount of experimental information about genes, proteins and biochemical reactions involved in regulating the cell division cycle of *Caulobacter crescentus*. These molecular details are spread over many specific publications and have not been drawn together into a coherent quantitative model until recently [24],[25]. Within the framework of nonlinear ordinary differential equations, we have developed a realistic, quantitative mathematical model of the molecular machinery governing the asymmetric division cycle of *Caulobacter*. Our model provides an opportunity to study and analyze the system-level dynamics of the *Caulobacter* cell cycle (beyond the kinetics of individual biochemical reactions), in order to test hypotheses about molecular mechanisms in silico, and to suggest new experimental designs.

The process of building a computational model is itself a scientifically challenging problem because it involves integrating data from diverse sources, reconciling observations made by different researchers, identifying gaps in our knowledge, hypothesizing mechanisms to fill these gaps, and converting the comprehensive descriptive scheme into a quantitative model capable of generating accurate temporal dynamics of protein fluctuations and physiological events. Our success in building such a model indicates that there is already a “critical mass” of experimental data and theoretical ideas available to hypothesize a reasonable mechanism controlling cell division in *Caulobacter*. Since our knowledge is incomplete, since any model inevitably contains many assumptions, and since there are alternative ways to express reaction kinetics in mathematical form, the proposed molecular mechanism and mathematical implementation of the model must be considered as an evolving hypothesis to be continually examined, revised, and improved as new observations tell us more about the molecular regulatory system.

Confidence in the model and respect for its predictions depend on how realistic is the underlying molecular mechanism, how accurate are the mathematical representations of the reaction network, and how well are the kinetic parameters of the model constrained by experimental data. Our model, consisting of 28 differential equations (Table S1) with 96 parameters (Table S2), was fitted to available experimental data, including phenotypes of wild-type and mutant cells, evidences for specific regulatory interactions, and quantitative measurements of protein expression. From the parameter list, the degradation rate constants of proteins (18 of 55 *k'*s) were determined from experimental data in the literature. Four *P'*s, which are relative gene positions on the chromosome, were estimated from the *Caulobacter* genome sequence. The production rate constants of components (37 of 55 *k'*s), together with 31 *J'*s and 6 *θ's* (binding constants and thresholds), were estimated by fitting the model to data points of 28 components in wild-type cells and by further tuning the parameter values to account for the phenotypes of 33 known mutants. By carefully fitting parameter values to experimental data wherever possible, we have constructed a model that captures the dynamics of cell cycle control in wild type (stalked and swarmer) cells as well as the phenotypic characteristics of numerous mutant strains. The model predicts a large amount of phenotypic properties not previously reported in known mutants, as well as phenotypes of 7 totally novel mutants.

Cell cycle regulation in *Caulobacter* is known to utilize spatial as well as temporal controls [47],[81],[82]. Spatial aspects of the control system are left for future versions of the model. As to the time domain, our model has limitations of temporal resolution and duration of a simulation. Details of simulations at the scale of 10 min or less do not necessarily have biological significance. On the other end, we can be reasonably confident of simulations of non-cycling cells up to about 400 min of run-time; for later times the model may exhibit artifacts or completely break down.

*Caulobacter crescentus* has recently been detected as a human pathogen, which makes the study of *Caulobacter* reproduction directly related to human health. Since many genes and mechanisms discovered in *Caulobacter* are evolutionarily conserved among other α-proteobacteria, our computational model of cell replication in *Caulobacter* may be extendable to other family members, in particular to the causative agents of brucellosis in cattle and Rocky Mountain spotted fever in humans. Insights gained into the temporal and spatial control of gene expression and protein interactions in *Caulobacter* could provide new clues for rational design of antibacterial agents and bacterial-based drug delivery technologies.

## Materials and Methods

### Scope of the Model

The model presented in this publication describes temporal regulation of the asymmetric cell division cycle in *Caulobacter crescentus*. It accounts for cell cycle regulation in both swarmer and stalked cells. In the former case, the model includes G1 (swarmer-to-stalked cell transition), S, and G2/M phases, while only S and G2/M phases are present for the stalked cell cycle. The regulatory mechanism is described at the protein level with addition of a few phenomenological variables to describe DNA replication and cytokinesis. Justification of our approach may be found in [23],[25].

At the core of the model are three master regulators (DnaA, GcrA, and CtrA), as in our previous publication [25]. Fluctuations of CtrA activity have been refined here to include explicitly protein activation via CckA-mediated phosphorylation of CtrA, and regulated degradation of CtrA by CpdR, RcdA, and ClpXP. Z-ring formation is described now by a separate variable, and its formation and closing are regulated by FtsZ and FtsQ proteins, which are described here as distinct variables, and by DNA replication. Finally, to capture regulation of the swarmer-to-stalked cell transition (differentiation), we have extended the model to include DivJ, PleC, PodJ, and PerP proteins. A complete list of the genes and proteins considered in this manuscript is provided in Table S5.

### The Quantitative Mathematical Model

Based on our model assumptions (Text S1), the interaction network for cell cycle controls in *Caulobacter* (Figure S1) summarizes the current stage of knowledge compiled from the literature. The wiring diagram in Figure 2 projects the interactions of Figure S1 into ‘protein space’ [83] and focuses on their relationships with physiological events [4]. The molecular mechanism is converted into a set of differential equations (Supplemental Table S1) describing the production, degradation, activation, inhibition, binding, release, phosphorylation, dephosphorylation, localization and delocalization of these proteins and physiological variables. The current model consists of 28 equations presented in Table S1, including 55 kinetic constants (*k's*), 31 binding constants (*J's*), and 6 thresholds (*θ's*). The choice of parameter values and initial conditions are given in Supplemental Tables S2 and S3.

Equations of the model were solved numerically with Matlab 2007b (The MathWorks). An online simulator and machine-readable files for reproducing our simulations are made available in Text S2 and on our website (http://mpf.biol.vt.edu/research/caulobacter/SWST/pp/).

### Parameter Values and Initial Conditions

Parameter values for our model (Table S2) were determined directly from experimental data, where possible, or chosen as in [25] to provide a good fit to available observations of wild type cells (see Supplemental Figure S2) and of mutant cells. We do not assert that this set of parameter values is optimal in any sense. Initial conditions in Table S3 were taken to represent the beginning of DNA replication whether it is for simulating a stalked cell cycle or swarmer cell cycle, in wild-type cells or mutants.

### Simulation of Mutants

The phenotypes of relevant mutants were collected from the literature. These mutants were simulated, as described in [25], using exactly the same equations (Table S1) and parameter values (Table S2) except for values of those parameters directly affected by the mutation (Table S4). In most cases, mutations are introduced in our model at the beginning of the swarmer cell, after 120 min of simulation.

## Supporting Information

Figure S1 Known cell cycle genes and their regulatory network in C. crescentus. Adapted from [25]. The circle portion at the right denotes the protein localization process involved into CtrA proteolysis. The gray rectangle at the center represents the concentration gradient of DivK phosphorylation during the cell cycle.
(1.04 MB TIF)

Figure S2 Comparison of simulated protein time profiles and DNA accumulation (curves) with experimental data (points).
(1.31 MB TIF)

Text S1 Assumptions of the Model
(0.07 MB DOC)

Text S2 Matlab Code
(0.09 MB DOC)

Table S1 Equations of the Model
(0.08 MB DOC)

Table S2 Parameter Values (Wild Type)
(0.22 MB DOC)

Table S3 Initial Conditions
(0.03 MB DOC)

Table S4 Parameter Changes (Mutants)
(0.09 MB DOC)

Table S5 Gene and proteins used in the paper
(0.05 MB DOC)
