# Supplementary figures and images for: Temporal Controls of the Asymmetric Cell Division Cycle in Caulobacter crescentus
Source: PLoS Comput Biol. 2009 Aug 14;5(8):e1000463. doi: 10.1371/journal.pcbi.1000463 (PMC2714070; doi:10.1371/journal.pcbi.1000463)

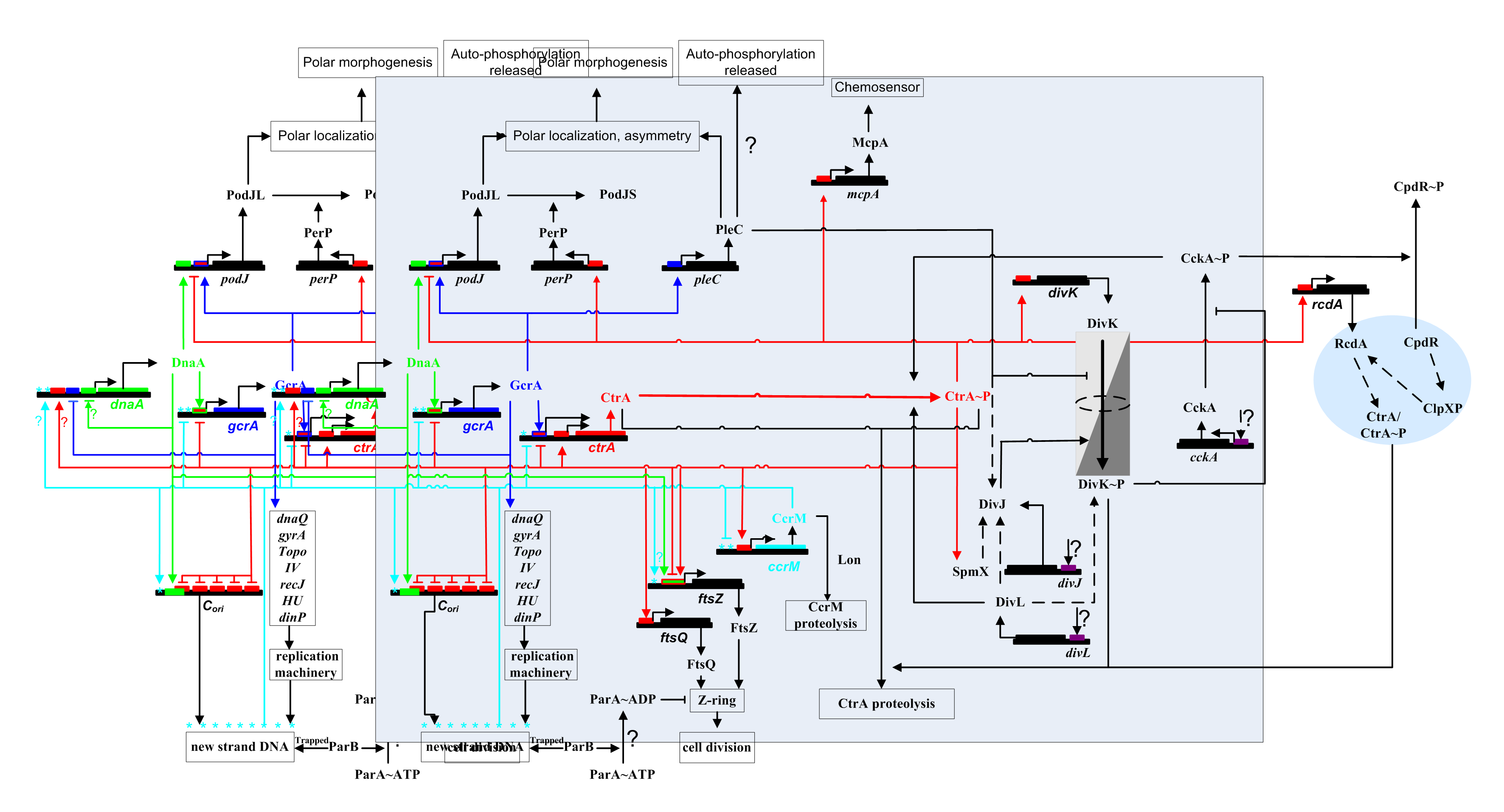

Supplement: Figure S1 — Known cell cycle genes and their regulatory network in C. crescentus. Adapted from [25]. The circle portion at the right denotes the protein localization process involved into CtrA proteolysis. The gray rectangle at the center represents the concentration gradient of DivK phosphorylation during the cell cycle. (1.04 MB TIF) [file pcbi.1000463.s001.tif]

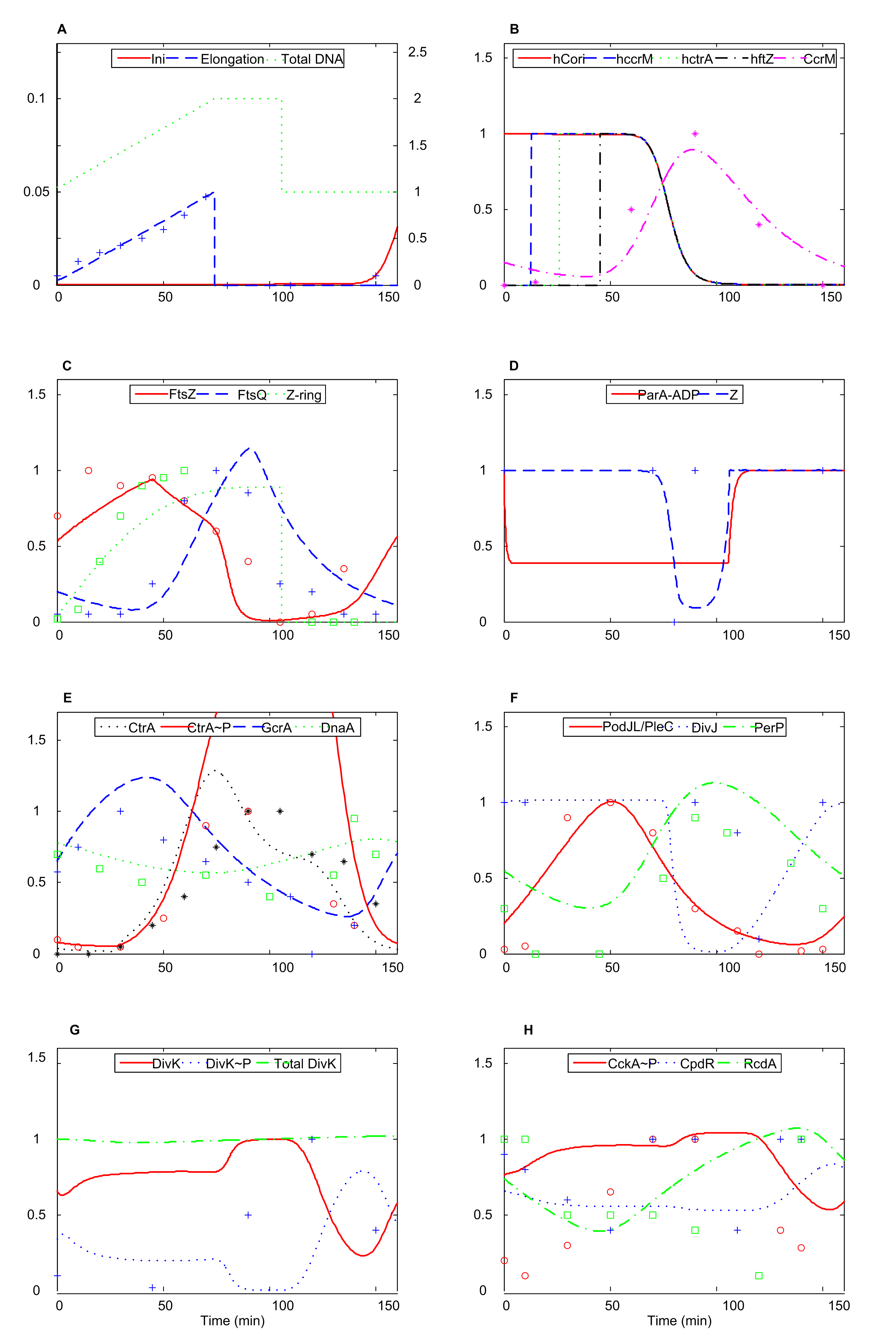

Supplement: Figure S2 — Comparison of simulated protein time profiles and DNA accumulation (curves) with experimental data (points). (1.31 MB TIF) [file pcbi.1000463.s002.tif]
