# Supplementary material for: Temporal Controls of the Asymmetric Cell Division Cycle in Caulobacter crescentus
Source: PLoS Comput Biol. 2009 Aug 14;5(8):e1000463. doi: 10.1371/journal.pcbi.1000463 (PMC2714070; doi:10.1371/journal.pcbi.1000463)
Supplement: Text S1 — Assumptions of the Model (0.07 MB DOC) [file pcbi.1000463.s003.doc]

Assumptions of the Model

Major assumptions made in the process of building our model are listed below. Some technical assumptions of the mathematical model are described also in Table 1.

1. As before [1], we model average behavior of cells and do not address naturally occurring, stochastic fluctuations in cell cycle progression.
2. Phosphorylation of CtrA is known to be regulated by CckA, DivL and some other proteins [2]. However, how these regulators are produced, degraded, localized, controlled, and details of their involvement in CtrA phosphorylation are not clear. Therefore we choose CckA as a representative protein promoting CtrA phosphorylation. The total amount of CckA is observed to be constant through the cell cycle, except for a sharp peak of expression during the swarmer-to-stalked cell transition, triggered by as yet unknown signals [3]. Consequently, in our model we keep total amount of CckA protein constant and track only variations of its active form, CckA~P, caused by the inhibitory action of DivK~P.

1. CtrA~P is widely accepted as the active form of the transcription factor that regulates expression of many genes [4,5]. However, it is not entirely clear from the literature whether gene expressions are regulated by CtrA~P only or by both CtrA and CtrA~P. In our model we assume that CtrA~P is the only active form of the protein.
2. RcdA, CpdR and ClpXP contribute to the degradation of CtrA and CtrA~P by a complex process including their own localization and recruiting CtrA to the stalked cell pole for proteolysis [6]. DivK~P was proposed to be an important signal determining when CtrA proteolysis happens [6]. In this version of the model, we complemented this proposal with effects of CpdR and RcdA on CtrA degradation, assuming that ClpXP is always in abundance. As is generally the case in this model, the localization of proteins involved in CtrA degradation is not being represented explicitly. In those cases where protein localization is essential to its function, the variable in our model represents the localized (active) form of the protein. Finally, for lack of any evidence to the contrary, CtrA and CtrA~P are assumed here to share the same degradation pathways (nonspecific and DivK-regulated).
3. In our previous model [1], the effects of proteins of the Fts family on Z ring formation and constriction were lumped together under a phenomenological variable called Fts. Here we replace ‘Fts’ with concentrations of proteins FtsZ and FtsQ, whose time variations during cell cycle are tuned against available experimental time-courses. Effects of the methylation states of the *ftsQ* and *ftsZ* genes are also accounted for explicitly in this version of the model. Since the *ftsQ* gene is expressed only when the chromosome is being duplicated [7], we assume that FtsQ is produced only when the origin of replication is duplicated and in the hemimethylated state (*h*_Cori_=1). The total decrease of FtsZ protein is assumed to be due to a background rate of degradation, rapid degradation after Z-ring constriction, and removal during Z-ring assembly. We do not model explicitly in the current model effects of other members of the Fts family like FtsA, FtsI, etc.
4. In contrast to our previous model, here we consider assembly and constriction of the Z-ring separately. The [Zring] variable represents the progress of Z ring assembly (from 0 to 1), and the [Z] variable represents the state of constriction ([Z] = 0 means fully constricted). Two factors are assumed to contribute to Z-ring assembly: the abundance of Z-ring building blocks (FtsZ protein, and a checkpoint signal from DNA replication needed to initiate Z-ring assembly correctly in the mid-cell plane [8]. In our model, the Z-ring disappears ([Zring] is reset to zero) immediately after full constriction. The conditions for Z-ring constriction are: an abundance of FtsQ, and a lack of ParA-ADP.
5. CckA, DivJ and PodJ_L_/PleC are some of the many Caulobacter proteins whose activity depends on spatial localization at specific phase of the cell cycle [9,10,11]. In this model, we do not propose to follow spatial redistribution of these proteins during the cell cycle. Instead, we use ODEs to describe how their functional activities vary during the cell cycle, assuming that each one is appropriately localized when its ‘activity’ is high.
6. PodJ, PleC, DivJ and PerP proteins are involved in the swarmer-to-stalked cell transition, and recently yet another protein, SpmX, was reported to be involved in this process [12]. Details of how these proteins collaborate in the transition are not clear. Therefore, for simplicity, we consider at this stage PodJ_L_ and PleC as a single state variable (PodJ_L_/PleC) which is responsible for dephosphorylation of DivK~P. In this scheme, PodJ_L_ is truncated to a shorter form by PerP during the swarmer-to-stalked cell transition, causing PodJ_L_/PleC to be replaced by DivJ at the pole as it changes from the flagellum to the stalk. DivJ phosphorylated DivK and DivK~P then degrades CtrA~P, completing the transition to the stalked cell state. At cell division, PodJ_L_/PleC and DivJ are separated into progeny swarmer and stalked cells respectively. The parameter *H* (see Table 1) determines which progeny cell the model will track (*H* = 1 for high DivJ activity in the stalked cell, or *H* = 0 for high PodJ_L_/PleC activity in the swarmer cell).
7. We assume that the total amounts of CckA, CpdR and ParA are constants, and only their transformations between active and inactive forms are modeled.

References

1. Li S, Brazhnik P, Sobral B, Tyson JJ (2008) A quantitative study of the division cycle of Caulobacter crescentus stalked cells. PLoS Comput Biol 4: e9.

2. Ausmees N, Jacobs-Wagner C (2003) Spatial and temporal control of differentiation and cell cycle progression in Caulobacter crescentus. AnnuRevMicrobiol 57: 225-247.

3. Jacobs C, Domian IJ, Maddock JR, Shapiro L (1999) Cell cycle-dependent polar localization of an essential bacterial histidine kinase that controls DNA replication and cell division. Cell 97: 111-120.

4. Holtzendorff J, Reinhardt J, Viollier PH (2006) Cell cycle control by oscillating regulatory proteins in Caulobacter crescentus. Bioessays 28: 355-361.

5. Jacobs C, Ausmees N, Cordwell SJ, Shapiro L, Laub MT (2003) Functions of the CckA histidine kinase in Caulobacter cell cycle control. MolMicrobiol 47: 1279-1290.

6. McGrath PT, Iniesta AA, Ryan KR, Shapiro L, McAdams HH (2006) A dynamically localized protease complex and a polar specificity factor control a cell cycle master regulator. Cell 124: 535-547.

7. Wortinger M, Sackett MJ, Brun YV (2000) CtrA mediates a DNA replication checkpoint that prevents cell division in Caulobacter crescentus. Embo Journal 19: 4503-4512.

8. Easter J, Gober JW (2002) ParB-stimulated nucleotide exchange regulates a switch in functionally distinct ParA activities. Molecular Cell 10: 427-434.

9. Laub MT, Shapiro L, McAdams HH (2007) Systems biology of Caulobacter. Annu Rev Genet 41: 429-441.

10. Goley ED, Iniesta AA, Shapiro L (2007) Cell cycle regulation in Caulobacter: location, location, location. J Cell Sci 120: 3501-3507.

11. Ebersbach G, Jacobs-Wagner C (2007) Exploration into the spatial and temporal mechanisms of bacterial polarity. Trends Microbiol 15: 101-108.

12. Radhakrishnan SK, Thanbichler M, Viollier PH (2008) The dynamic interplay between a cell fate determinant and a lysozyme homolog drives the asymmetric division cycle of Caulobacter crescentus. Genes Dev 22: 212-225.
