# Supplementary material for: Temporal Controls of the Asymmetric Cell Division Cycle in Caulobacter crescentus
Source: PLoS Comput Biol. 2009 Aug 14;5(8):e1000463. doi: 10.1371/journal.pcbi.1000463 (PMC2714070; doi:10.1371/journal.pcbi.1000463)
Supplement: Text S2 — Matlab Code (0.09 MB DOC) [file pcbi.1000463.s004.doc]

CauloSTSM.m File

function Results = CauloSTSM(celltype)

%%&&&&&&&&&&&&&&&&&&&&&&&&&&&&&&&&&&&&&&&&&&&&&&&&&&&&&&&&&&&&&&&&&&&&&&&&%

% Script for numericaly simulation of Caulobacter cell cycle model

% Sep. 9, 2008. Shenghua Li et al.

% This script needs 'events.m' and 'modelODEs.m'

% for running.

%&&&&&&&&&&&&&&&&&&&&&&&&&&&&&&&&&&&&&&&&&&&&&&&&&&&&&&&&&&&&&&&&&&&&&&&&&%

% celltype determines which cell cycle (swarmer or stalked) we are tracking.

if nargin < 1

celltype = 0;

end

if celltype == 'stalked'

celltype = 1;

elseif celltype == 'swarmer'

celltype = 0;

else

celltype = 0;

end

% global variable CELLTYPE is used to send the celltype information to

% 'swstmodelwtsimin.m' which is the equations input.

global CELLTYPE;

CELLTYPE = celltype;

%&&&&&&&&&&&&&&&&&&&&&&&&&&&&&&&&&&&&&&&&&&&&&&&&&&&&&&&&&&&&&&&&&&&&&&&&&%

%%variable map

% DnaA, GcrA, CtrA, CtrA~P

%y(1) = [DnaA]

%y(2) = [GcrA]

%y(3) = [CtrA]

%y(4) = [CtrA~P]

%y(5) = 0

% DivK, DivK~P, total DivK

%y(6) = [DivK]

%y(7) = [DivK~P]

%y(8) = [Total DivK]

% DNA replication, methylation

%y(9) = [I] (Intermediate)]

%y(10) = [CcrM]

%y(11) = [hCori] (hemimethylated Cori)

%y(12) = [hccrM] (hemimethylated ccrM)

%y(13) = [hctrA] (hemimethylated ctrA)

%y(14) = [hfts](hemimethylated fts)

%y(15) = [Ini] (Initiation)

%y(16) = [Elong](Elongation)

%y(17) = [DNA] (Total DNA)

%y(18) = Count

% PodJL/PleC, PerP, DivJ

%y(19) = PodJL/PleC

%y(20) = PerP

%y(21) = DivJ

% RcdA, CpdR, CckA~P

%y(32) = CckA~P

%y(33) = CpdR

%y(34) = RcdA

%y(34) = 0

%y(35) = 0

% FtsZ, FtsQ, Ring, Zring

%y(36) = Z (Z-ring constriction)

%y(37) = 0

%y(38) = 0

%y(39) = ParA-ADP

%y(40) = Z-ring (Z-ring assembly)

%y(41) = FtsZ

%y(42) = FtsQ

%&&&&&&&&&&&&&&&&&&&&&&&&&&&&&&&&&&&&&&&&&&&&&&&&&&&&&&&&&&&&&&&&%&&&&&&&&%

% Initial value of model variables, for a newborn, wild-type stalked cell

y0 = zeros(42, 1);

y0(1) = 0.78;

y0(2) = 0.65;

y0(3) = 0.04;

y0(4) = 0.08;

y0(5) = 0.81;

y0(6) = 0.66;

y0(7) = 0.34;

y0(8) = 1.0;

y0(9) = 0.09;

y0(10) = 0.15;

y0(11) = 1;

y0(12) = 0;

y0(13) = 0;

y0(14) = 0;

y0(15) = 0.0;

y0(16) = 0.05;

y0(17) = 1.05;

y0(18) = 2;

y0(19) = 0.2;

y0(20) = 0.55;

y0(21) = 1.0;

y0(22) = 0.18;

y0(23) = 0.82;

y0(24) = 1.0;

y0(25) = 1.0;

y0(26) = 1.0;

y0(27) = 0.63;

y0(28) = 0.37;

y0(29) = 0.9;

y0(30) = 0.44;

y0(31) = 0.6;

y0(32) = 0.76;

y0(33) = 0.66;

y0(34) = 0.74;

y0(35) = 0.26;

y0(36) = 1.0;

y0(37) = 1.0;

y0(38) = 1.0;

y0(39) = 1.0;

y0(40) = 0.04;

y0(41) = 0.53;

y0(42) =0.2;

% end of initial values

%&&&&&&&&&&&&&&&&&&&&&&&&&&&&&&&&&&&&&&&&&&&&&&&&&&&&&&&&&&&&&&&&&&&&&&&&%

% Integration parameters

tspan = [0 470];

tstart = tspan(1);

tfinal = tspan(2);

options = odeset('Events', @events, 'RelTol', 1e-5, 'AbsTol', 1e-6);

% end of integration parameters

%&&&&&&&&&&&&&&&&&&&&&&&&&&&&&&&&&&&&&&&&&&&&&&&&&&&&&&&&&&&&&&&&&&&&&&&&%

% Numerical Integration

tout = tstart;

yout = y0.';

teout = [];

yeout = [];

ieout = [];

% loop for continous simulation of the differential equations.

% i is used to counted the interrupt points through the simulation. It can be

% changed to larger to satisfy longer time simulation once more interrupt

% points are detected.

while tstart < tfinal

[T, Y, TE, YE, IE] = ode15s(@modelODEs, [tstart tfinal], y0, options);

% Accumulate output.

nt = length(T);

tout = [tout;T(2:nt)];

yout = [yout;Y(2:nt, :)];

teout = [teout;TE];

yeout = [yeout;YE];

ieout = [ieout;IE];

% Refersh the initial conditions for differential equations once the interrupt

% point is detected.

y0 = Y(nt, :);

if isscalar(IE) == 0

IE = 0;

end

switch (IE)

case 1

y0(16) = Y(nt, 16) + Y(nt, 15);

y0(17) = Y(nt, 17) + Y(nt, 15);

y0(18) = Y(nt, 18)*2;

y0(15) = 0;

y0(11) = 1;

y0(38) = 1;

case 2

y0(12) = Y(nt, 12) + 1;

case 3

y0(13) = Y(nt, 13) + 1;

case 4

y0(14) = Y(nt, 14) + 1;

case 5

y0(16) = 0;

case 6

y0(16) = Y(nt, 16)/2;

y0(17) = Y(nt, 17)/2;

y0(18) = Y(nt, 18)/2;

y0(24) = 1;

y0(40) = 0;

y0(38) = 0;

end

tstart = T(nt);

if tstart >= tfinal

break;

end

end

%end of integration

%&&&&&&&&&&&&&&&&&&&&&&&&&&&&&&&&&&&&&&&&&&&&&&&&&&&&&&&&&&&&&&&&&&&&&&&&&&%

%% Save the data

%save('swstmodelwtsim30.txt', 'tout', 'yout', '-ASCII');

%Save the initial data at t = 270 min

%y_initial = interp1(tout, yout, 442);

%save('swstmodelwtsim445.txt', 'y_initial', '-ASCII');

%&&&&&&&&&&&&&&&&&&&&&&&&&&&&&&&&&&&&&&&&&&&&&&&&&&&&&&&&&&&&&&&&&&&&&&&&&&%

close all;

%&&&&&&&&&&&&&&&&&&&&&&&&&&&&&&&&&&&&&&&&&&&&&&&&&&&&&&&&&&&&&&&&&&&&&&&&&&%

%% Ploting the time courses of model variables

figure(1);

set(gcf, 'Name', 'Numerical Simulation of Model for Caulobacter Cell Cycle');

set(gcf, 'Outerposition', [0 0 1280 1024]);

subplot(2, 2, 1)

p1 = line(tout, yout(:, 15), 'Color', 'r', 'LineWidth', 2, 'Linestyle', '-');

axis([tspan 0 0.13]);

ax1 = gca;

set(ax1, 'XTick', 0:50:tspan(2), 'YTick', 0:0.05:0.13, 'Fontsize', 9);

ylabel('A', 'Rotation', 0);

ax2 = axes('Position', get(ax1, 'Position'), 'YAxisLocation', 'right', 'Color', 'none', 'Box', 'on');

p2 = line(tout, yout(:, 16), 'Color', 'b', 'LineWidth', 2, 'Linestyle', '--');

axis([tspan 0 2.6]);

set(ax2, 'XTick', 0:50:tspan(2), 'YTick', 0:0.5:2.6, 'XTickLabel', '', 'Fontsize', 9);

p3 = line(tout, yout(:, 17), 'Color', 'g', 'LineWidth', 2, 'Linestyle', ':');

axis([tspan 0 2.6]);

h = legend([p1, p2, p3], 'Ini', 'Elongation', 'Total DNA', 'Location', 'North');

set(h, 'Interpreter', 'none', 'Box', 'on', 'Orientation', 'horizontal');

subplot(2, 2, 2)

p1 = line(tout, yout(:, 11), 'Color', 'r', 'LineWidth', 2, 'Linestyle', '-');

p2 = line(tout, yout(:, 12), 'Color', 'b', 'LineWidth', 2, 'Linestyle', '--');

p3 = line(tout, yout(:, 13), 'Color', 'g', 'LineWidth', 2, 'Linestyle', ':');

p4 = line(tout, yout(:, 14), 'Color', 'k', 'LineWidth', 2, 'Linestyle', '-.');

p5 = line(tout, yout(:, 10), 'Color', 'm', 'LineWidth', 2, 'Linestyle', '-.');

axis([tspan 0 1.6]);

ax1 = gca;

set(ax1, 'XTick', tspan(1):50:tspan(2), 'YTick', 0:0.5:1.6, 'Fontsize', 9, 'Box', 'on');

h = legend('hCori', 'hccrM', 'hctrA', 'hftZ', 'CcrM', 'Location', 'North');

set(h, 'Interpreter', 'none', 'Box', 'on', 'Orientation', 'horizontal');

ylabel('B', 'Rotation', 0);

subplot(2, 2, 3)

p1 = line(tout, yout(:, 41), 'Color', 'r', 'LineWidth', 2, 'Linestyle', '-');

p2 = line(tout, yout(:, 42), 'Color', 'b', 'LineWidth', 2, 'Linestyle', '--');

p3 = line(tout, yout(:, 40), 'Color', 'g', 'LineWidth', 2, 'Linestyle', ':');

axis([tspan 0 1.6]);

ax1 = gca;

set(ax1, 'XTick', tspan(1):50:tspan(2), 'YTick', 0:0.5:1.6, 'Fontsize', 9, 'Box', 'on');

h = legend('FtsZ', 'FtsQ', 'Ring', 'Location', 'North');

set(h, 'Interpreter', 'none', 'Box', 'on', 'Orientation', 'horizontal');

ylabel('C', 'Rotation', 0);

subplot(2, 2, 4)

p1 = line(tout, yout(:, 39), 'Color', 'r', 'LineWidth', 2, 'Linestyle', '-');

p2 = line(tout, yout(:, 36), 'Color', 'b', 'LineWidth', 2, 'Linestyle', '--');

axis([tspan 0 1.6]);

ax1 = gca;

set(ax1, 'XTick', tspan(1):50:tspan(2), 'YTick', 0:0.5:1.6, 'Fontsize', 9, 'Box', 'on');

h = legend('ParA-ADP', 'Z', 'Location', 'North');

set(h, 'Interpreter', 'none', 'Box', 'on', 'Orientation', 'horizontal');

ylabel('D', 'Rotation', 0);

figure(2);

set(gcf, 'Name', 'Numerical Simulation of Model for Caulobacter Cell Cycle');

set(gcf, 'Outerposition', [0 0 1280 1024]);

subplot(2, 2, 1)

p1 = line(tout, yout(:, 3), 'Color', 'k', 'LineWidth', 2, 'Linestyle', ':');

p2 = line(tout, yout(:, 4), 'Color', 'r', 'LineWidth', 2, 'Linestyle', '-');

p3 = line(tout, yout(:, 2), 'Color', 'b', 'LineWidth', 2, 'Linestyle', '--');

p4 = line(tout, yout(:, 1), 'Color', 'g', 'LineWidth', 2, 'Linestyle', ':');

axis([tspan 0 1.7]);

ax1 = gca;

set(ax1, 'XTick', tspan(1):50:tspan(2), 'YTick', 0:0.5:1.7, 'Fontsize', 9, 'Box', 'on');

h = legend('CtrA', 'CtrA~P', 'GcrA', 'DnaA', 'Location', 'North');

set(h, 'Interpreter', 'none', 'Box', 'on', 'Orientation', 'horizontal');

ylabel('E', 'Rotation', 0);

subplot(2, 2, 2)

p1 = line(tout, yout(:, 19), 'Color', 'r', 'LineWidth', 2, 'Linestyle', '-');

p2 = line(tout, yout(:, 21), 'Color', 'b', 'LineWidth', 2, 'Linestyle', ':');

p3 = line(tout, yout(:, 20), 'Color', 'g', 'LineWidth', 2, 'Linestyle', '-.');

axis([tspan 0 1.6]);

ax1 = gca;

set(ax1, 'XTick', tspan(1):50:tspan(2), 'YTick', 0:0.5:1.6, 'Fontsize', 9, 'Box', 'on');

h = legend('PodJL/PleC', 'DivJ', 'PerP', 'Location', 'North');

set(h, 'Interpreter', 'none', 'Box', 'on', 'Orientation', 'horizontal');

ylabel('F', 'Rotation', 0);

subplot(2, 2, 3)

p1 = line(tout, yout(:, 6), 'Color', 'r', 'LineWidth', 2, 'Linestyle', '-');

p2 = line(tout, yout(:, 7), 'Color', 'b', 'LineWidth', 2, 'Linestyle', ':');

p3 = line(tout, yout(:, 8), 'Color', 'g', 'LineWidth', 2, 'Linestyle', '-.');

axis([tspan 0 1.6]);

ax1 = gca;

set(ax1, 'XTick', tspan(1):50:tspan(2), 'YTick', 0:0.5:1.6, 'Fontsize', 9, 'Box', 'on');

xlabel('Time (min)', 'FontSize', 11);

h = legend('DivK', 'DivK~P', 'Total DivK', 'Location', 'North');

set(h, 'Interpreter', 'none', 'Box', 'on', 'Orientation', 'horizontal');

ylabel('G', 'Rotation', 0);

subplot(2, 2, 4)

p1 = line(tout, yout(:, 32), 'Color', 'r', 'LineWidth', 2, 'Linestyle', '-');

p2 = line(tout, yout(:, 33), 'Color', 'b', 'LineWidth', 2, 'Linestyle', ':');

p3 = line(tout, yout(:, 34), 'Color', 'g', 'LineWidth', 2, 'Linestyle', '-.');

axis([tspan 0 1.6]);

ax1 = gca;

set(ax1, 'XTick', tspan(1):50:tspan(2), 'YTick', 0:0.5:1.6, 'Fontsize', 9, 'Box', 'on');

xlabel('Time (min)', 'FontSize', 11);

h = legend('CckA~P', 'CpdR', 'RcdA', 'Location', 'North');

set(h, 'Interpreter', 'none', 'Box', 'on', 'Orientation', 'horizontal');

ylabel('H', 'Rotation', 0);

%end of plotting

%&&&&&&&&&&&&&&&&&&&&&&&&&&&&&&&&&& &&&&&&&&&&&&&&&&&&&&&&&&&&&&&&&&&&&&&&%

% clear all;

clc;

end

modelODEs.m File

function dydt = modelODEs(t, y)

%%&&&&&&&&&&&&&&&&&&&&&&&&&&&&&&&&&&&&&&&&&&&&&&&&&&&&&&&&&&&&&&&&&&&&&&&&%

% The input equations of model for wild-type cells

% Sep. 9, 2008. Shenghua Li et al.

%&&&&&&&&&&&&&&&&&&&&&&&&&&&&&&&&&&&&&&&&&&&&&&&&&&&&&&&&&&&&&&&&%&&&&&&&&%

%%variable map

% DnaA, GcrA, CtrA, CtrA~P

%y(1) = [DnaA]

%y(2) = [GcrA]

%y(3) = [CtrA]

%y(4) = [CtrA~P]

%y(5) = 0

% DivK, DivK~P, total DivK

%y(6) = [DivK]

%y(7) = [DivK~P]

%y(8) = [Total DivK]

% DNA replication, methylation

%y(9) = [I] (Intermediate)]

%y(10) = [CcrM]

%y(11) = [hCori] (hemimethylated Cori)

%y(12) = [hccrM] (hemimethylated ccrM)

%y(13) = [hctrA] (hemimethylated ctrA)

%y(14) = [hfts](hemimethylated fts)

%y(15) = [Ini] (Initiation)

%y(16) = [Elong](Elongation)

%y(17) = [DNA] (Total DNA)

%y(18) = Count

% PodJL/PleC, PerP, DivJ

%y(19) = PodJL/PleC

%y(20) = PerP

%y(21) = DivJ

% RcdA, CpdR, CckA~P

%y(32) = CckA~P

%y(33) = CpdR

%y(34) = RcdA

%y(34) = 0

%y(35) = 0

% FtsZ, FtsQ, Ring, Zring

%y(36) = Z (Z-ring constriction)

%y(37) = 0

%y(38) = 0

%y(39) = ParA-ADP

%y(40) = Z-ring (Z-ring assembly)

%y(41) = FtsZ

%y(42) = FtsQ

%&&&&&&&&&&&&&&&&&&&&&&&&&&&&&&&&&&&&&&&&&&&&&&&&&&&&&&&&&&&&&&&&%&&&&&&&&%

% parameters for which cell we are tracking on

global CELLTYPE;

% cell type track

H = CELLTYPE;

%H = 1;

% parameters for the equations

% DnaA, GcrA, CtrA, CtrA~P

ksDnaA1 = 0.0031;

JiDnaAGcrA = 0.6; niDnaAGcrA = 2;

JiDnaAGcrA2 = 0.8;

ksDnaA2 = 0.0022;

JaDnaACtrA = 0.3; naDnaACtrA = 2;

JaDnaACtrA2 = 0.7;

kdDnaA = 0.007;

ksDnaA = 0.0075;

ksGcrA = 0.055; JiGcrACtrA = 0.4; niGcrACtrA = 2;

kdGcrA = 0.022;

ksCtrAP1 = 0.0083*2.4*0.8; JiCtrACtrA = 0.4; niCtrACtrA = 2;

ksCtrAP2 = 0.073*2.4*0.8; JaCtrACtrA = 0.45; naCtrACtrA = 2;

kdCtrA1 = 0.002;

kdCtrA2 = 0.25; ndCtrA2 = 2;

JdCtrADivKP = 0.55;

JdCtrARcdA = 0.5; ndCtrARcdA = 4;

ndCtrACpdR = 4;

JdCtrACpdR = 0.6;

%ktransCtrAP = 0.025; ktransCtrA = 0.205;

ktransCtrAP = 0.025; ktransCtrA = 0.095;

% DNA replication, methylation

kaIni = 0.01; JaIni = 1; naIni = 4;

thetaCtrA = 0.5; nthetaCtrA = 4;

thetaDnaA = 0.65; nthetaDnaA = 4;

thetaGcrA = 0.65; nthetaGcrA = 4;

thetaCori = 0.05; nthetaCori = 4;

kelong = 0.95/160*1.1;

nelong = 4;

ksI = 0.09;

JaICtrA = 0.5;

naICtrA = 2;

kdI = 0.04;

ksCcrM = 0.072; kdCcrM = 0.07;

kmcori = 0.4; Jmcori = 0.95; nmcori = 4;

kmccrM = 0.4; JmccrM = 0.95; nmccrM = 4;

kmctrA = 0.4; JmctrA = 0.95; nmctrA = 4;

kmftsZ = 0.4; JmftsZ = 0.95; nmftsZ = 4;

% PodJ/PleC, PerP, DivJ

ksPodJL = 0.043; JiPodJLCtrA = 0.6; niPodJLCtrA = 2;

kd1PodJL = 0.05; JiPodJLPerP = 0.45; niPodJLPerP = 2;

kd2PodJL = 0.002;

ksepPodJL = 0.3; JsepPodJL = 0.3;

ksPerP = 0.04; kdPerP = 0.02;

JaPerPCtrA = 0.1;

ksepPerP = 0.011; JsepPerP = 0.3;

ksDivJ1 = 0.002;

ksDivJ2 = 0.025; JiDivJPodJL = 0.13; niDivJPodJL = 4;

kdDivJ = 0.002;

ksepDivJ = 0.3; JsepDivJ = 0.3;

nsep = 4;

% DivK, DivK~P

ksDivK = 0.0024; JaDivKCtrA = 0.06; naDivKCtrA = 2; kdDivK = 0.002;

ktransDivKP = 0.6; ktransDivK = 0.15;

JDivKPPodJL = 0.3; nDivKPPodJL = 2;

JDivKDivJ = 0.3; nDivKDivJ = 2;

% ParA

ktransParAATP = 0.5;

ktransParAADP = 0.8;

ParAtot = 1.0;

%RcdA, CpdR, CckA~P

ksRcdA = 0.023; kdRcdA = 0.017;

JaRcdACtrA = 0.4; naRcdACtrA = 2;

ktransCpdRP = 0.5;

ktransCpdR = 0.6;

JdCpdRCckAP = 0.8;

nCpdRCckAP = 4;

CpdRtot = 1.0;

ktransCckAP = 0.05; ktransCckA = 0.2;

JiCckADivKP = 0.3; niCckADivKP = 2;

CckAtot = 1.3;

% FtsZ, FtsQ, Z-ring, Z

kZopen = 0.8; JaZopen = 0.01;

kZclosed1 = 0.0001;

JaZclose = 0.05;

JZFtsQ = 0.8;

nZclosed2 = 4;

kZclosed2 = 1.6; nZclosed3 = 4;

JZRing = 0.3;

JZParAADP = 0.3;

kZring = 0.035;

JFtsZFtsQ = 0.1;

nFtsZFtsQ = 4;

ksFtsZ = 0.036; kdFtsZ1 = 0.009;

kdFtsZ2 = 0.02;

kdFtsZ3 = 0.3;

JiFtsZCtrA = 0.7; niFtsZCtrA = 2;

ksFtsQ = 0.06;

kdFtsQ = 0.035;

JiFtsQCtrA = 0.5;

niFtsQCtrA = 2;

naFtsQDNA = 4;

JaFtsQDNA = 0.05;

naFtsQDNA = 4;

JaFtsQDNA = 0.05;

ksFtsZ2 = 0.036; kdFtsZ1 = 0.009;

kdFtsZ22 = 0.02;

kdFtsZ33 = 0.3;

% end of parameters

%&&&&&&&&&&&&&&&&&&&&&&&&&&&&&&&&&&&&&&&&&&&&&&&&&&&&&&&&&&&&&&&&%&&&&&&&&%

% This section is used for simulations of mutants

%if t > 120

% kop = (0.0083 + 0.073)*1.5;

% ksCtrAP1 = 0;

% ksCtrAP2 = 0;

%end

%&&&&&&&&&&&&&&&&&&&&&&&&&&&&&&&&&&&&&&&&&&&&&&&&&&&&&&&&&&&&&&&&%&&&&&&&&%

% Differential equations of the model

dydt = zeros(42, 1);

% DnaA, GcrA, CtrA, CtrA~P

dydt(1) = (ksDnaA1*JiDnaAGcrA^niDnaAGcrA/(JiDnaAGcrA^niDnaAGcrA + y(2)^niDnaAGcrA) + ksDnaA2*y(4)^naDnaACtrA/(JaDnaACtrA^naDnaACtrA + y(4)^naDnaACtrA))*(2 - y(11)) - kdDnaA*y(1);

dydt(2) = (ksGcrA*JiGcrACtrA^niGcrACtrA/(JiGcrACtrA^niGcrACtrA + y(4)^niGcrACtrA)*y(1) - kdGcrA*y(2));

dydt(3) = (ksCtrAP1*JiCtrACtrA^niCtrACtrA/(JiCtrACtrA^niCtrACtrA + y(4)^niCtrACtrA)*y(2) + ksCtrAP2*y(4)^naCtrACtrA/(JaCtrACtrA^naCtrACtrA + y(4)^naCtrACtrA))*y(13) - (kdCtrA1 + kdCtrA2*y(7)^ndCtrA2/(JdCtrADivKP^ndCtrA2 + y(7)^ndCtrA2)*y(33)^ndCtrACpdR/(JdCtrACpdR^ndCtrACpdR + y(33)^ndCtrACpdR)*y(34)^ndCtrARcdA/(JdCtrARcdA^ndCtrARcdA + y(34)^ndCtrARcdA))*y(3) + ktransCtrAP*y(4) - ktransCtrA*y(3)*y(32);

dydt(4) = - (kdCtrA1 + kdCtrA2*y(7)^ndCtrA2/(JdCtrADivKP^ndCtrA2 + y(7)^ndCtrA2)*y(33)^ndCtrACpdR/(JdCtrACpdR^ndCtrACpdR + y(33)^ndCtrACpdR)*y(34)^ndCtrARcdA/(JdCtrARcdA^ndCtrARcdA + y(34)^ndCtrARcdA))*y(4) - ktransCtrAP*y(4) + ktransCtrA*y(3)*y(32);

dydt(5) = 0;

% DivK, DivK~P, total DivK

dydt(6) = (ksDivK*y(4)^naDivKCtrA/(JaDivKCtrA^2 + y(4)^naDivKCtrA) + ktransDivKP*y(7)*y(19)^nDivKPPodJL/(y(19)^nDivKPPodJL + JDivKPPodJL^nDivKPPodJL)*(1 + H*(y(36) - 1)) - ktransDivK*y(6)*y(21)^2/(y(21)^2 + JDivKDivJ^2)*(y(36) + H*(1 - y(36))) - kdDivK*y(6));

dydt(7) = ( - ktransDivKP*y(7)*y(19)^nDivKPPodJL/(y(19)^nDivKPPodJL + JDivKPPodJL^nDivKPPodJL)*(1 + H*(y(36) - 1)) + ktransDivK*y(6)*y(21)^nDivKDivJ/(y(21)^nDivKDivJ + JDivKDivJ^nDivKDivJ)*(y(36) + H*(1 - y(36))) - kdDivK*y(7));

dydt(8) = ksDivK*y(4)^naDivKCtrA/(JaDivKCtrA^2 + y(4)^naDivKCtrA) - kdDivK*y(8);

%CcrM and DNA methylation

dydt(9) = ksI*y(12)*y(4)^naICtrA/(JaICtrA^2 + y(4)^naICtrA) - kdI*y(9);

dydt(10) = ksCcrM*y(9) - kdCcrM*y(10);

dydt(11) = - kmcori*y(10)^nmcori/(Jmcori^nmcori + y(10)^nmcori)*y(11);

dydt(12) = - kmccrM*y(10)^nmccrM/(JmccrM^nmccrM + y(10)^nmccrM)*y(12);

dydt(13) = - kmctrA*y(10)^nmctrA/(JmctrA^nmctrA + y(10)^nmctrA)*y(13);

dydt(14) = - kmftsZ*y(10)^nmftsZ/(JmftsZ^nmftsZ + y(10)^nmftsZ)*y(14);

%DNA replication

dydt(15) = kaIni*(y(1)/thetaDnaA)^nthetaDnaA*(y(2)/thetaGcrA)^nthetaGcrA/(JaIni^naIni + (y(4)/thetaCtrA)^nthetaCtrA + (y(1)/thetaDnaA)^nthetaDnaA + (y(2)/thetaGcrA)^nthetaGcrA + (y(1)/thetaDnaA)^nthetaDnaA*(y(2)/thetaGcrA)^nthetaGcrA + (y(11)/thetaCori)^nthetaCori)*y(18);

dydt(16) = kelong*y(16)^nelong/(y(16)^nelong + 0.05^nelong)*y(18);

dydt(17) = kelong*y(16)^nelong/(y(16)^nelong + 0.05^nelong)*y(18);

dydt(18) = 0;

%PodJ, PerP, PleC and DivJ

dydt(19) = ksPodJL*JiPodJLCtrA^niPodJLCtrA/(JiPodJLCtrA^niPodJLCtrA + y(4)^niPodJLCtrA)*y(2)*y(1) - kd1PodJL*y(20)^niPodJLPerP/(y(20)^niPodJLPerP + JiPodJLPerP^niPodJLPerP)*y(19) - kd2PodJL*y(19) - ksepPodJL*y(19)*H*(1 - y(36))^nsep/(JsepPodJL^nsep + (1 - y(36))^nsep);

dydt(20) = ksPerP*y(19)*y(4) - kdPerP*y(20) - ksepPerP*y(20)*H*(1 - y(36))^nsep/(JsepPerP^nsep + (1 - y(36))^nsep);

dydt(21) = ksDivJ1 + (1 - H)*ksDivJ2*JiDivJPodJL^niDivJPodJL/(JiDivJPodJL^niDivJPodJL + y(19)^niDivJPodJL) - kdDivJ*y(21) - ksepDivJ*y(21)*(1 - H)*(1 - y(36))^nsep/(JsepDivJ^nsep + (1 - y(36))^nsep);

%RcdA, CpdR, ClpXP, CckA

dydt(22) = 0;

dydt(23) = 0;

dydt(24) = 0;

dydt(25) = 0;

dydt(26) = 0;

dydt(27) = 0;

dydt(28) = 0;

dydt(29) = 0;

dydt(30) = 0;

dydt(31) = 0;

dydt(32) = - ktransCckAP*y(32) + ktransCckA*(CckAtot - y(32))*JiCckADivKP^niCckADivKP/(JiCckADivKP^niCckADivKP + y(7)^niCckADivKP);

dydt(33) = ktransCpdRP*(CpdRtot - y(33)) - ktransCpdR*y(33)*y(32)^nCpdRCckAP/(JdCpdRCckAP^nCpdRCckAP + y(32)^nCpdRCckAP);

dydt(34) = ksRcdA*y(4)^naRcdACtrA/(JaRcdACtrA^naRcdACtrA + y(4)^naRcdACtrA) - kdRcdA*y(34);

%FtsZ, FtsQ, Z-ring, Z

dydt(35) = 0;

dydt(36) = (kZopen*(1 - y(36))/(JaZopen + (1 - y(36))) - (kZclosed1 + kZclosed2*y(42)^nZclosed2/(y(42)^nZclosed2 + JZFtsQ^nZclosed2)*(y(40)/JZRing)^nZclosed3/(1 + (y(40)/JZRing)^nZclosed3 + (y(39)/JZParAADP)^nZclosed3))*y(36)/(JaZclose + y(36)));

dydt(37) = 0;

dydt(38) = 0;

dydt(39) = ktransParAATP*(ParAtot - y(39)) - ktransParAADP*(y(18) - 1)*y(39);

dydt(40) = kZring*y(41)*(1 - y(40))*y(38);

dydt(41) = ksFtsZ*JiFtsZCtrA^niFtsZCtrA/(JiFtsZCtrA^niFtsZCtrA + y(4)^niFtsZCtrA)*y(1)*(1 - y(14)) - (kdFtsZ1 + kdFtsZ2*(1 - y(40))*y(11) + kdFtsZ3* (1 - y(36)))*y(41);

dydt(42) = ksFtsQ*y(4)^niFtsQCtrA/(JiFtsQCtrA^niFtsQCtrA + y(4)^niFtsQCtrA)*y(11)^naFtsQDNA/(y(11)^naFtsQDNA + JaFtsQDNA^naFtsQDNA) - kdFtsQ*y(42);

% end of equations

%&&&&&&&&&&&&&&&&&&&&&&&&&&&&&&&&&&&&&&&&&&&&&&&&&&&&&&&&&&&&&&&&%&&&&&&&&%

end

events.m Figle

%&&&&&&&&&&&&&&&&&&&&&&&&&&&&&&&&&&&&&&&&&&&&&&&&&&&&&&&&&&&&&&&&&&&&&&&&&&%

% This is the script for detecting specific events of the cell cycle.

% Sep. 09, 2008. Shenghua Li et al.

% This script is required by 'CauloModel.m' for running.

%&&&&&&&&&&&&&&&&&&&&&&&&&&&&&&&&&&&&&&&&&&&&&&&&&&&&&&&&&&&&&&&&&&&&&&&&&&%

function [value,isterminal,direction] = events(t, y)

% Locate the time when values passes through zero in a

% increasing direction and stop integration.

value=[sign(y(15) - 0.05*y(18)); sign(2*y(16) - 0.2*y(18)); sign(2*y(16) - 0.375*y(18)); sign(2*y(16) - 0.625*y(18)); sign(2*y(16) - y(18)); sign(y(36) - 0.7)];

isterminal=[1; 1; 1; 1; 1; 1; 1];

direction=[+1; +1; +1; +1; +1; +1];

%&&&&&&&&&&&&&&&&&&&&&&&&&&&&&&&&&&&&&&&&&&&&&&&&&&&&&&&&&&&&&&&&&&&&&&&&&&%
