# Supplementary material for: Temporal Controls of the Asymmetric Cell Division Cycle in Caulobacter crescentus
Source: PLoS Comput Biol. 2009 Aug 14;5(8):e1000463. doi: 10.1371/journal.pcbi.1000463 (PMC2714070; doi:10.1371/journal.pcbi.1000463)
Supplement: Table S1 — Equations of the Model (0.08 MB DOC) [file pcbi.1000463.s005.doc]

**Table S1. Equations of the Model**

$$\begin{matrix} \text{\# The choice of what cell cycle is being tracked} \\ \\ \text{(1) For stalked cell }H\text{ = 1; for swarmer cell }H\text{ = 0. } \\ \\ \text{\# Three major regulators : DnaA, GcrA, CtrA/CtrA\textasciitilde P} \\ \\ (2)\text{ }\frac{\text{d[CtrA]}}{\text{dt}}\text{ = }\left(k_{\text{s,CtrA-P1}}\frac{J_{\text{i,CtrA-CtrA\textasciitilde P}}^{2}}{J_{\text{i,CtrA-CtrA\textasciitilde P}}^{2}+\text{[CtrA\textasciitilde P]}^{2}}\cdot\text{[GcrA]}+k_{\text{s,CtrA-P2}}\frac{\text{[CtrA\textasciitilde P]}^{2}}{J_{\text{a,CtrA-CtrA\textasciitilde P}}^{2}+\text{[CtrA\textasciitilde P]}^{2}}\right)\cdot\text{[h}_{\text{ctrA}}\text{]} \\ \text{ }-\left(k_{\text{d,CtrA1}}+k_{\text{d,CtrA2}}\frac{\text{[DivK\textasciitilde P]}^{\text{ 2}}}{J_{\text{d,CtrA-DivK\textasciitilde P}}^{2}+\text{[DivK\textasciitilde P]}^{\text{ 2}}}\cdot\frac{\text{[CpdR]}^{\text{ 4}}}{J_{\text{d,CtrA-CpdR}}^{4}+\text{[CpdR]}^{\text{ 4}}}\cdot\frac{\text{[RcdA]}^{\text{ 4}}}{J_{\text{d,CtrA-RcdA}}^{4}+\text{[RcdA]}^{\text{ 4}}}\right)\cdot\text{[CtrA]} \\ \text{ }+k_{\text{trans,CtrA\textasciitilde P}}\cdot\text{[CtrA\textasciitilde P]}-k_{\text{trans,CtrA}}\cdot\text{[CtrA]}\cdot\text{[CckA\textasciitilde P]} \\ \\ (3)\text{ }\frac{\text{d[CtrA\textasciitilde P]}}{\text{dt}}\text{ = }-\left(k_{\text{d,CtrA1}}+k_{\text{d,CtrA2}}\frac{\text{[DivK\textasciitilde P]}^{\text{ 2}}}{J_{\text{d,CtrA-DivK\textasciitilde P}}^{2}+\text{[DivK\textasciitilde P]}^{\text{ 2}}}\cdot\frac{\text{[CpdR]}^{\text{ 4}}}{J_{\text{d,CtrA-CpdR}}^{4}+\text{[CpdR]}^{\text{ 4}}}\cdot\frac{\text{[RcdA]}^{\text{ 4}}}{J_{\text{d,CtrA-RcdA}}^{4}+\text{[RcdA]}^{\text{ 4}}}\right)\cdot\text{[CtrA\textasciitilde P]} \\ \text{ }-k_{\text{trans,CtrA\textasciitilde P}}\cdot\text{[CtrA\textasciitilde P]}+k_{\text{trans,CtrA}}\cdot\text{[CtrA]}\cdot\text{[CckA\textasciitilde P]} \\ \\ (4)\text{ }\frac{\text{d[DnaA]}}{\text{dt}}\text{ = }\left(k_{\text{s,DnaA1}}\cdot\frac{J_{\text{i,DnaA-GcrA}}^{2}}{J_{\text{i,DnaA-GcrA}}^{2}+\text{[GcrA]}^{2}}+k_{\text{s,DnaA2}}\cdot\frac{\text{[CtrA\textasciitilde P]}^{2}}{J_{\text{a,DnaA-CtrA\textasciitilde P}}^{2}+\text{[CtrA\textasciitilde P]}^{2}}\right)\cdot\left(2-\text{[h}_{\text{cori}}\text{]}\right)-k_{\text{d,DnaA}}\cdot\text{[DnaA]} \\ \\ (5)\text{ }\frac{\text{d[GcrA]}}{\text{dt}}\text{ = }k_{\text{s,GcrA}}\cdot\frac{J_{\text{i,GcrA-CtrA\textasciitilde P}}^{2}}{J_{\text{i,GcrA-CtrA}}^{2}+\text{[CtrA\textasciitilde P]}^{2}}\cdot\text{[DnaA]}-k_{\text{d,GcrA}}\cdot\text{[GcrA]} \end{matrix}$$

$$\begin{matrix} \text{\# RcdA, CckA\textasciitilde P, CpdR, and ParA-ADP} \\ \\ (21)\text{ }\frac{\text{d[CckA\textasciitilde P]}}{\text{dt}}\text{ = }-k_{\text{trans,CckA\textasciitilde P}}\cdot\text{[CckA\textasciitilde P]}+k_{\text{trans,CckA}}\cdot\left(\text{[CckA]}_{\text{tot}}-\text{[CckA]}\right)\cdot\frac{J_{\text{i,CckA-DivK\textasciitilde P}}^{2}}{J_{\text{i,CckA-DivK\textasciitilde P}}^{2}+\text{[DivK\textasciitilde P]}^{\text{ 2}}} \\ \\ (22)\text{ }\frac{\text{d[CpdR]}}{\text{dt}}\text{ = }k_{\text{trans,CpdR\textasciitilde P}}\cdot\left(\text{[CpdR]}_{\text{tot}}-\text{[CpdR]}\right)-k_{\text{trans,CpdR}}\cdot\text{[CpdR]}\cdot\frac{\text{[CckA\textasciitilde P]}^{2}}{J_{\text{a,CpdR-CckA\textasciitilde P}}^{2}+\text{[CckA\textasciitilde P]}^{\text{ 2}}}\text{ } \\ \\ (23)\text{ }\frac{\text{d[ParAADP]}}{\text{dt}}\text{ = }k_{\text{trans,ParAATP}}\cdot\left(\text{[ParA]}_{\text{tot}}-\text{[ParAADP]}\right)-k_{\text{trans,ParAADP}}\cdot\left(Count-1\right)\cdot\text{[ParAADP]} \\ \\ (24)\text{ }\frac{\text{d[RcdA]}}{\text{dt}}\text{ = }k_{\text{s,RcdA}}\cdot\frac{\text{[CtrA\textasciitilde P]}^{2}}{J_{\text{a,RcdA-CtrA\textasciitilde P}}^{2}+\text{[CtrA\textasciitilde P]}^{\text{ 2}}}-\text{ }k_{\text{d,RcdA}}\cdot\text{[RcdA]} \end{matrix}$$

$$\begin{matrix} \#\text{ Z-ring formation, constriction} \\ \\ (25)\text{ }\frac{\text{d[FtsQ]}}{\text{dt}}\text{ = }k_{\text{s,FtsQ}}\cdot\frac{\text{[CtrA\textasciitilde P]}^{2}}{J_{\text{a, FtsQ-CtrA\textasciitilde P}}^{2}+\text{[CtrA\textasciitilde P]}^{2}}\cdot\frac{\text{[h}_{\text{Cori}}\text{]}^{4}}{J_{\text{FtsQ-DNA}}^{4}+\text{[h}_{\text{Cori}}\text{]}^{4}}-k_{\text{d,FtsQ}}\cdot\text{[FtsQ]} \\ \text{Note that FtsQ is produced only when DNA is in duplication, which is adjusted here by [h}_{\text{Cori}}\text{] term.} \\ \\ (26)\text{ }\frac{\text{d[FtsZ]}}{\text{dt}}\text{ = }k_{\text{s,FtsZ}}\cdot\frac{J_{\text{i, FtsZ-CtrA\textasciitilde P}}^{2}}{J_{\text{i, FtsZ-CtrA\textasciitilde P}}^{2}+\text{[CtrA\textasciitilde P]}^{2}}\cdot\text{[DnaA]}\cdot\left(1-\text{[h}_{\text{ftsZ}}\text{]}\right)-\left(k_{\text{d,FtsZ1}}+k_{\text{d,FtsZ2}}\cdot\left(1-\text{[Zing]}\right)+k_{\text{d,FtsZ3}}\cdot\left(1-\text{[Z]}\right)\right)\cdot\text{[FtsZ]} \\ \\ \text{ [Zring] is the phenomenological variable describing assembly of the Z-ring.} \\ \text{ In the begining of the cycle [Zring] = 0. } \\ \text{ [Zring] grows (Z-ring forms) when there is FtsZ available.} \\ \text{ When Z-ring constricts completely ([Z] = 0), [Zring] is reset to zero.} \\ (27)\text{ }\frac{\text{d[Zring]}}{\text{dt}}\text{ = }k_{\text{s,Zring}}\cdot\left(1-\text{[Zring]}\right)\cdot\text{[FtsZ]} \\ \\ \text{ [Z] is the phenomenological variable describing closure of the Z ring. } \\ \text{ [Z] = 1 when the Z ring is open, in the begining of the cycle, and [Z] = 0 when it is fully constricted.} \\ (28)\text{ }\frac{\text{d[Z]}}{\text{dt}}\text{ = }k_{\text{Z,open}}\cdot\frac{1-\text{[Z]}}{\text{J}_{\text{a,open}}+1-\text{[Z]}}-\left(k_{\text{Z,closed1}}+k_{\text{Z,closed2}}\cdot\frac{\text{[FtsQ]}^{4}}{J_{\text{a, Z-FtsQ}}^{4}+\text{[FtsQ]}^{4}}\cdot\frac{\left(\text{[Zing]/}\theta_{\text{Zring}}\right)^{4}}{1+\left(\text{[Zing]/}\theta_{\text{Zring}}\right)^{4}+\left(\text{[ParAADP]/}\theta_{\text{ParAADP}}\right)^{4}}\right)\cdot\frac{\text{[Z]}}{J_{\text{a,closed}}+\text{[Z]}} \end{matrix}$$

Symbols:

*k* = rate constants (min^-1^), *J* = binding constants (dimensionless), *θ* = thresholds (dimensionless), *P* = position of genes relative to origin site (dimensionless). s = synthesis, d = degradation, a = activation, i = inactivation, trans = transformation (phosphorylation and dephosphorylation in our case), m = methylation, sep = separation due to Z-ring constriction, tot = total amount of proteins.
