# Supplementary material for: Temporal Controls of the Asymmetric Cell Division Cycle in Caulobacter crescentus
Source: PLoS Comput Biol. 2009 Aug 14;5(8):e1000463. doi: 10.1371/journal.pcbi.1000463 (PMC2714070; doi:10.1371/journal.pcbi.1000463)
Supplement: Table S2 — Parameter Values (Wild Type) (0.22 MB DOC) [file pcbi.1000463.s006.doc]

**Table S2: Basal parameter values for the wild-type cell division cycle.**

| A. Rate constants, units = min^-1^ | | | | |
| --- | --- | --- | --- | --- |
| *k*_s,CtrA-P1_ = 0.0159, *k*_s,CtrA-P2_ = 0.14 | | | (Domian et al., 1999; Grunenfelder et al., 2001; Holtzendorff et al., 2004; Jacobs et al., 2003) | |
| *k*_d,CtrA1_ = 0.002 | | | (Hung and Shapiro, 2002) | |
| *k*_d,CtrA2_ = 0.25 | | | (Domian et al., 1997) | |
| *k*_trans,CtrA~P_ = 0.025 | | | (Jacobs et al., 2003) | |
| *k*_trans,CtrA_ = 0.095 | | | (Jacobs et al., 2003) | |
| *k*_s,GcrA_ = 0.055 | | | (Holtzendorff et al., 2004) | |
| *k*_d,GcrA_ = 0.022 | | | (Collier et al., 2006; Holtzendorff et al., 2004) | |
| *k*_s,DnaA1_ = 0.0031, *k*_s,DnaA2_ = 0.0022 | | | (Collier et al., 2007; Collier et al., 2006; Zweiger and Shapiro, 1994) | |
| *k*_d,DnaA_ = 0.007 | | | (Gorbatyuk and Marczynski, 2005) | |
| *k*_a,Ini_ = 0.01 | | | (Quon et al., 1998) | |
| *k*_elong_ = 0.0065 | | | (Dingwall and Shapiro, 1989) | |
| *k*_s,I_ = 0.09, *k*_d,I_ = 0.04 | | | (Grunenfelder et al., 2001) | |
| *k*_s,CcrM_ = 0.072 | | | (Grunenfelder et al., 2001) | |
| *k*_d,CcrM_ = 0.07 | | | (Stephens et al., 1996) | |
| *k*_m,Cori_ = 0.4, *k*_m,ctrA_ = 0.4 | | | (Stephens et al., 1996) | |
| *k*_m,ccrM_ = 0.4, *k*_m,ftsZ_ = 0.4 | | | (Stephens et al., 1996) | |
| *k*_s,PodJL_ = 0.043 | | | (Chen et al., 2006; Viollier et al., 2002b) | |
| *k*_d,PodJL1_ = 0.05, *k*_d,PodJL2_ = 0.002 | | | (Chen et al., 2006; Viollier et al., 2002b) | |
| *k*_sep,PodJL_ = 0.3 | | | (Chen et al., 2006; Viollier et al., 2002b) | |
| *k*_s,PerP_ = 0.04, *k*_d,PerP_ = 0.02 | | | (Chen et al., 2006) | |
| *k*_sep,PerP_ = 0.011 | | | (Chen et al., 2006) | |
| *k*_s,DivJ1_ = 0.002 | | | (Wheeler and Shapiro, 1999) | |
| *k*_s,DivJ2_ = 0.025 | | | (Jacobs et al., 2001) | |
| *k*_d,DivJ_ = 0.002 | | | (Wheeler and Shapiro, 1999) | |
| *k*_sep,divJ_ = 0.3 | | | (Jacobs et al., 2001; Pierce et al., 2006) | |
| *k*_s,DivK_ = 0.0024 | | | (Grunenfelder et al., 2001) | |
| *k*_d,DivK_ = 0.002, *k*_d,DivK~P_ = 0.002 | | | (Jacobs et al., 2001) | |
| *k*_trans,DivK_ = 0.15, *k*_trans,DivK~P_ = 0.6 | | | (Jacobs et al., 2001) | |
| *k*_trans,CckA~P_ = 0.05, *k*_trans,CckA_ = 0.2 | | | (Iniesta et al., 2006; Jacobs et al., 2003) | |
| *k*_trans,CpdR_ = 0.6, *k*_trans,CpdR~P_ = 0.5 | | | (Iniesta et al., 2006) | |
| *k*_trans,ParAATP_ = 0.5, *k*_trans,ParAADP_ = 0.8 | | | (Figge et al., 2003) | |
| *k*_s,RcdA_ = 0.023, *k*_d,RcdA_ = 0.017 | | | (McGrath et al., 2006) | |
| *k*_s,FtsQ_ = 0.1, *k*_d,FtsQ_ = 0.035 | | | (Martin et al., 2004; Sackett et al., 1998) | |
| *k*_s,FtsZ_ = 0.036 | | | (Martin et al., 2004; Sackett et al., 1998) | |
| *k*_d,FtsZ1_ = 0.009, *k*_d,FtsZ2_ = 0.02 | | | (Martin et al., 2004; Sackett et al., 1998) | |
| *k*_d,FtsZ3_ = 0.3 | | | (Aaron et al., 2007) | |
| *k*_s,Zring_ = 0.035 | | | (Aaron et al., 2007) | |
| *k*_Z,open_ = 0.8 | | | (Judd et al., 2003) | |
| *k*_Z,closed1_ = 0.0001, *k*_Z,closed2_ = 1.6 | | | (Judd et al., 2003) | |
| B. Binding constants and thresholds (dimensionless) | | | | |
| *J*_i,CtrA-CtrA~P_ = 0.4 | *J*_a,CtrA-CtrA~P_ = 0.45 | *J*_d,CtrA-DivK~P_ = 0.55 | | *J*_d,CtrA-CpdR_ = 0.6 |
| *J*_d,CtrA-RcdA_ = 0.5 | *J*_i,GcrA-CtrA_ = 0.4 | *J*_i,DnaA-GcrA_ = 0.6 | | *J*_a,DnaA-CtrA~P_ = 0.3 |
| *J*_a,I-CtrA~P_ = 0.5 | *J*_m,Cori_ = 0.95 | *J*_m,ctrA_ = 0.95 | | *J*_m,ccrM_ = 0.95 |
| *J*_m,ftsZ_ = 0.95 | *J*_i,PodJL-CtrA~P_ = 0.6 | *J*_d,PodJL-PerP_ = 0.45 | | *J*_sep, PodJL_ = 0.3 |
| *J*_sep, PerP_ = 0.3 | *J*_i,DivJ-PodJL_ = 0.13 | *J*_sep, DivJ_ = 0.3 | | *J*_a, DivK_ = 0.06 |
| *J*_DivK~P-PodJL_ = 0.3 | *J*_DivK-DivJ_ = 0.3 | *J*_i, CckA-DivK~P_ = 0.3 | | *J*_a, CpdR-CckA~P_ = 0.8 |
| *J*_a, RcdA-CtrA~P_ = 0.4 | *J*_a, FtsQ-CtrA~P_ = 0.5 | *J*_FtsQ, DNA_ = 0.4 | | *J*_i, FtsZ-CtrA~P_ = 0.7 |
| J_a,open_ = 0.01 | *J*_Z-FtsQ_ = 0.8 | *J*_a,closed_ = 0.05 | |  |
| *θ*_CtrA~P_ = 0.5 | *θ*_GcrA_ = 0.65 | *θ*_DnaA_ = 0.65 | | *θ*_Cori_ = 0.05 |
| *θ*_Zring_ = 0.3 | *θ*_ParAADP_ = 0.3 |  | |  |
| C. Gene positions on the chromosome (dimensionless, from [http://ecocyc.org](http://ecocyc.org/)) | | | | |
| *P*_elong_^a^ = 0.05 | *P*_ccrM_ = 0.2 | *P*_ctrA_ = 0.375 | | *P*_ftsZ_ = 0.625 |
| *^a^P*_elong_ is assumed to be the end point of replication initiation and the starting point of chromosome elongation. | | | | |
