# Supplementary material for: Temporal Controls of the Asymmetric Cell Division Cycle in Caulobacter crescentus
Source: PLoS Comput Biol. 2009 Aug 14;5(8):e1000463. doi: 10.1371/journal.pcbi.1000463 (PMC2714070; doi:10.1371/journal.pcbi.1000463)
Supplement: Table S3 — Initial Conditions (0.03 MB DOC) [file pcbi.1000463.s007.doc]

**Table S3: Initial values of model variables for a newborn, wild-type stalked cell**

| [DnaA] = 0.78 | [GcrA] = 0.65 | [CtrA] = 0.04 | [CtrA] = 0.08 |
| --- | --- | --- | --- |
| [DivK] = 0.66 | [DivK~P] = 0.34 | [I] = 0.09 | [CcrM] = 0.15 |
| [h_Cori_] = 1.0 | [h_ctrA_] = 0 | [h_ccrM_] = 0 | [h_ftsZ_] = 0 |
| [Ini] = 0.0 | [Elong] = 0.05 | [DNA] = 1.05 | Count = 2 |
| [PodJ_L_] = 0.2 | [PerP] = 0.55 | [DivJ] = 1.0 | [CckA~P] = 0.76 |
| [CpdR] = 0.66 | [RcdA] = 0.74 | [ParAADP] = 1.0 | [FtsZ] = 0.53 |
| [Zring] = 0.04 | [FtsQ] = 0.2 | [Z] = 1.0 | [*CckA*]*_tot_* = 1.3 |
| [*CpdR*]*_tot_* = 1.0 | [*ParA*]*_tot_* = 1.0 |  |  |
