# Supplementary material for: Temporal Controls of the Asymmetric Cell Division Cycle in Caulobacter crescentus
Source: PLoS Comput Biol. 2009 Aug 14;5(8):e1000463. doi: 10.1371/journal.pcbi.1000463 (PMC2714070; doi:10.1371/journal.pcbi.1000463)
Supplement: Table S4 — Parameter Changes (Mutants) (0.09 MB DOC) [file pcbi.1000463.s008.doc]

**Table S4: Altered parameter values for mutant simulations.**

| Genotype | Parameter changes* |
| --- | --- |
| ***ctrA* mutants** |  |
| *∆ctrA-*P1 | *k*_s,ctrA-P1_ = 0.0024 (15% of WT) |
| *∆ctrA-P2* | *k*_s,ctrA-P2_ = 0 |
| *∆ctrA* | *k*_s,ctrA-P1_ = *k*_s,ctrA-P2_ = 0 |
| *ctrA401^ts^* | *k*_s,ctrA-P1_, *k*_s,ctrA-P2_ : 75%, 40% and 10% of WT |
| *ctrAD51E* | *k*_s,ctrA-P1_ = *k*_s,ctrA-P2_ = 0,  *k*_trans,CtrA~P_ = 0,  *k*_trans,CtrA_ = 0  *k*’ = 0.064 was added to [CtrA~P] equation** |
| *ctrA*∆3Ω | *k*_d,ctrA2_ = 0.0375 (15% of WT) |
| *ctrAD51E*∆3Ω | *k*_s,ctrA-P1_ = *k*_s,ctrA-P2_ = 0,  *k*_trans,CtrA~P_ = 0, *k*_trans,CtrA_ = 0  *k*’ = 0.064 was added to [CtrA~P] equation  *k*_d,ctrA2_ = 0.0375 (15% of WT) |
| *ctrA* constit express | *k*_s,ctrA-P1_ = *k*_s,ctrA-P2_ = 0, *k*’ = 0.048 |
| *ctrA^op^ (P_xylX_-ctrA*∆3) | *k*’ = 0.16 |
| ***gcrA* mutants** |  |
| *∆gcrA* | *k*_s,GcrA_ = 0 |
| *gcrA^op^* | *k*’ = 0.0225 (50% of WT) or 0.0605 (110% of WT) |
| ***dnaA* mutants** |  |
| *∆dnaA* | *k*_s,DnaA_ = 0 |
| *∆dnaA* rescued | *k*_s,DnaA_ = 0.05 at 120 < t < 300 min  *k*_s,DnaA_ = 0.0 at 300 < t < 450 min  *k*_s,DnaA_ = 0.05 at t > 450 min |
| *dnaA^op^* | *k*’ = 0.0053 (100% of WT) |
| ***divK* mutants** |  |
| *∆divK* | *k*_s,DivK_ = 0 |
| *divK341^cs^* | *k*_d,ctrA2_ = 0 |
| *divK341^cs^* rescued | *k*_d,ctrA2_ = 0 at 120 < t < 190 min  *k*_d,ctrA2_ = 0.25 (100% of WT) at t > 190 min |
| *divK*_D53A_, etc. | *k*_trans,DivK_ = 0 |
| *divK^op^* | *k*’ = 0.024 (10 x WT) |
| ***ccrM* mutants** |  |
| *∆ccrM* | *k*_s,I_ = 0 |
| *ccrM^op^* | *k*’ = 0.09 |
| *Lon* null | *k*_d,CcrM_ = 0 |
| **DNA mutant** |  |
| *∆dnaC, dnaE^ts^* | *k*_elong_ = 0 |
| ***Fts* mutants** |  |
| *∆ftsQ* | *k*_s,FtsQ_ = 0 |
| *∆ftsZ* | *k*_s,FtsZ_ = 0 |
| ***parA* mutants** |  |
| *∆parA* | ParAtot = 0.5,  *k*_trans,ParA-ADP_ = 0.0 |
| *parA^op^* | ParAtot = 1.5,  *k*_trans,ParA-ADP_ = 0.0 |
| ***rcdA* mutants** |  |
| *∆rcdA* | *k*_s,RcdA_ = 0 |
| *∆rcdA + ctrAD51E* | *k*_s,RcdA_ = 0  *k*_s,ctrA-P1_ = *k*_s,ctrA-P2_ = 0,  *k*_trans,CtrA~P_ = 0,  *k*’ = 0.16 was added to [CtrA~P] equation |
| *rcdA^op^* | *k*’ = 0.023 |
| ***cckA* mutants** |  |
| *∆cckA (cckATS1)* | CckA_tot_ = 0.1 |
| CckA unphosph | *k*_trans,CckA_ = 0 |
| ***cpdR* mutants** |  |
| *∆cpdR* | CpdR_tot_ = 0.0 |
| *cpdRD51A* | *k*_trans,CpdR_ = 0 |
| *cpdR^op^* | CpdR_tot_ = 1.5 (50% overexpression of WT) |
| CpdR fully phosphorylated*** | *k*_trans,CpdRP_ = 0 |
| ***divJ* mutants** |  |
| *∆divJ* | *k*_s,DivJ1_ = *k*_s,DivJ2_ = 0 |
| *∆divJ + divK^op^* | *k*_s,DivJ1_ = 0.0014,  *k*_s,DivJ2_ = 0.0175 (70% of WT, can be lower or higher), *k*’ = 0.024 (10 x WT) |
| ***pleC/podJ* mutants** |  |
| *∆pleC* and *∆podJ* | *k*_s,PodJL_ = 0 |
| *pleC^op^* and *podJ^op^* | *k*_s,PodJL_ = 0,  *k*’ = 0.043 (100% of WT) |
| ***perP* mutants** |  |
| *∆perP* | *k*_s,PerP_ = 0 |
| *perP* const express | *k*_s,PerP_ = 0,  *k*’ = 0.04 (100% of WT) |

* *k*’ represents a constant rate of constitutive synthesis added to the relevant differential equation.

**Here *k*’ value is 40% of wild-type gene expression level. 10% ~ 60% level of wild-type gene expression shares the similar protein variations. If *k*’ value is below 10% of wild-type gene expression level, simulation results in *∆ctrA*. If *k*’ value is above 60% level of wild-type gene expression level, then simulation results in *ctrA^op^*.

***This mutant with fully methylated CpdR could be available through the replacement of specific amino acids (probably via point mutation of *cpdR* gene) to let CpdR be in full phosphorylation state whenever produced.
