# Supplementary material for: Temporal Controls of the Asymmetric Cell Division Cycle in Caulobacter crescentus
Source: PLoS Comput Biol. 2009 Aug 14;5(8):e1000463. doi: 10.1371/journal.pcbi.1000463 (PMC2714070; doi:10.1371/journal.pcbi.1000463)
Supplement: Table S5 — Gene and proteins used in the paper (0.05 MB DOC) [file pcbi.1000463.s009.doc]

**Table S5. Genes and proteins that appear in our manuscript**

|  | | |  |  |  |
| --- | --- | --- | --- | --- | --- |
| **CC#** | **GenBankName** | **GenBank Annotation** | **GenBankID** | **TIGR ORF #** | **EC #** |
| CC0008 | *dnaA* | chromosomal replication initiator protein DnaA | 13421094 | ORF02307 |  |
| CC0378 | *ccrM* | modification methylase CcrM | 13421535 | ORF03051 | 2.1.1.72 |
| CC0744 | *cpdR* | response regulator | 13421977 | ORF03749 |  |
| CC1063 | *divJ* | sensor histidine kinase DivJ | 13422363 | ORF04405 |  |
| CC1078 | *cckA* | cell cycle histidine kinase CckA | 13422380 | ORF04430 |  |
| CC1307 | *perP* | conserved hypothetical protein | 13422646 | ORF04859 |  |
| CC1960 | Lon | ATP-dependent protease LA | 13423421 | ORF06080 | 3.4.21.53 |
| CC1961 | *clpX* | ATP-dependent Clp protease, ATP-binding subunit ClpX | 13423422 | ORF06082 |  |
| CC1963 | *clpP* | ATP-dependent Clp protease, proteolytic subunit | 13423424 | ORF06085 | 3.4.21.92 |
| CC2045 | *podJ* | polar organelle development protein, authentic frameshift |  | ORF06244 |  |
| CC2245 | *gcrA* | hypothetical protein | 13423754 | ORF06648 |  |
| CC2463 | *divK* | polar differentiation response regulator | 13424012 | ORF07061 |  |
| CC2482 | *pleC* | non-motile and phage-resistance protein | 13424035 | ORF07098 |  |
| CC2540 | *ftsZ* | cell division protein FtsZ | 13424103 | ORF07201 |  |
| CC2541 | *ftsA* | cell division protein FtsA | 13424104 | ORF07204 |  |
| CC2542 | *ftsQ* | cell division protein FtsQ | 13424105 | ORF07205 |  |
| CC3035 | *ctrA* | cell cycle transcriptional regulator CtrA | 13424677 | ORF00839 |  |
| CC3295 | *rcdA* | hypothetical protein | 13424989 | ORF01381 |  |
| CC3752 | *parB* | chromosome partitioning protein ParB | 13425526 | ORF02270 |  |
| CC3753 | *parA* | chromosome partitioning protein ParA | 13425527 | ORF02271 |  |
| CC0909 | *flbD* | transcriptional regulator FlbD | 13422175 | ORF04083 |  |
| CC2165 | *mipZ* | conserved hypothetical protein | 13423660 | ORF06488 |  |
